# Phosphatidylcholine synthesis and remodeling in brain endothelial cells

**DOI:** 10.1016/j.jlr.2025.100773

**Published:** 2025-03-10

**Authors:** Mohamed H. Yaghmour, Theja Sajeevan, Christoph Thiele, Lars Kuerschner

**Affiliations:** LIMES Life and Medical Sciences Institute, University of Bonn, Bonn, Germany

**Keywords:** click, lysophosphatdiylcholine, lipid cycling, alkyne tracer, propargyl-PC, lipidomics, lysophospholipids, phospholipase

## Abstract

Mammalian cells synthesize hundreds of different variants of their prominent membrane lipid phosphatidylcholine (PC), all differing in the side chain composition. This batch is constantly remodeled by the Lands cycle, a metabolic pathway replacing one chain at a time. Using the alkyne lipid lyso-phosphatidylpropargylcholine (LpPC), a precursor and intermediate in PC synthesis and remodeling, we study both processes in brain endothelial bEND3 cells. A novel method for multiplexed sample analysis by mass spectrometry is developed that offers high throughput and molecular species resolution of the propargyl-labeled PC lipids. Their time-resolved profiles and kinetic parameters of metabolism demonstrate the plasticity of the PC pool and the acute handling of lipid influx in endothelial cells differs from that in hepatocytes. Side chain remodeling as a form of lipid cycling adapts the PC pool to the cell's need and maintains lipid homeostasis. We estimate that endothelial cells possess the theoretical capacity to remodel up to 99% of their PC pool within 3.5 h using the Lands cycle. However, PC species are not subjected stochastically to this remodeling pathway as different species containing duplets of saturated, omega-3, and omega-6 side chains show different decay kinetics. Our findings emphasize the essential function of Lands cycling for monitoring and adapting the side chain composition of PC in endothelial cells.

Eukaryotic cell membranes contain hundreds of different lipid species, and phosphatidylcholine (PC) comprises a major fraction thereof (1). Mammals in general synthesize PC by the Kennedy pathway (2), in addition by methylation of phosphatidylethanolamine in certain tissues (3), or as part of the Lands cycle (4). While through the two former pathways de novo synthesis is achieved, the latter is especially important for side chain remodeling. Newly synthesized PC molecules regularly contain a saturated and an unsaturated fatty acid (FA) at the *sn-1* and *sn-2* positions, respectively (5). Saturated palmitate or stearate mostly account for *sn-1* linked FAs, whereas oleate and linoleate are commonly found at the *sn-2* position upon de novo synthesis (4). To incorporate other FAs or adapt the PC pool to changing conditions the Lands cycle is employed. Upon liberation of the *sn-1* or *sn-2* linked FA by a phospholipase A_1_ or A_2_ (PLA_1/2_), respectively, the resulting lyso-phosphatidylcholine (LPC) is re-acylated by a lyso-phosphoplipid acyltransferase (LPLAT) using FA-CoA from the cellular pool (6). At least eight of the 14 known LPLATs acylate LPC (7). Determination of substrate specificity and kinetics has mostly been established on in vitro enzymatic activities (7). However, the selectivity for FAs as acylation partners observed in vitro is somewhat inconsistent with the specificity observed in vivo (8, 9). Rat liver predominantly uses tetraene and to a lesser extent diene FAs, but little monoene FAs (10), and no discrimination between omega-6 or omega-3 poly-unsaturated fatty acids (PUFAs) was found (11). In contrast, in vitro systems such as microsomal preparations or cell lysates show the usage of oleoyl-, linoleoyl-, and arachidonoyl-CoAs at comparable rates unless the amount of LPC becomes limiting (12). Under normal physiological conditions, there is a profound competition among all FAs for a limited number of esterification sites in vivo (13). Furthermore, the actual FA used during Lands cycling in a given tissue is not only determined by the substrate specificity of the locally expressed LPLATs but also by the relative abundance of the various FA-CoA species in that tissue (4). The latter is defined by endogenous synthesis levels, nutritional supply and rates of cellular uptake, thioester formation, and the activity of competing metabolic pathways.

Dietary and endogenously produced lipids are transported in the blood. The liver is a major lipid-secreting organ and serum albumin binds free FA and LPC while PC is a component of lipoprotein particles (14, 15, 16). Capillary endothelial cells expose lipases that facilitate lipid uptake from blood into the tissue (17). In fasted humans, the serum concentrations of FAs are 200–230 μM (18, 19), about twofold higher than those of LPCs although a range has been reported (18, 19, 20, 21). These concentrations vary pre- and postprandially and hence tissue cells are exposed to fluctuating levels of incoming lipids. Depending on the cell status the acquired lipids enter different metabolic pathways and may serve as catabolic substrates, are converted and stored as neutral lipids, or act as building blocks for membrane lipids such as PC.

To study PC metabolism, we have recently introduced a novel method based on click chemistry and a traceable alkyne analog of LPC, lyso-phosphatidylpropargylcholine (LpPC), where propargylcholine replaces the choline head group (22). Phosphatidylpropargylcholine (pPC) metabolism can be followed by microscopy (23, 24) and TLC using fluorogenic reporter azides (25, 26), or by MS using an azidopalmitate reporter (22). Hence, correlative data can be obtained (27) and lipid tracing by alkyne lipid probes has proven a powerful and valuable method (28).

The current study investigates PC synthesis and side chain remodeling in a brain endothelial cell line. For kinetic examinations, a large number of samples was analyzed triggering the development of a novel multiplexing method for the analysis of pPC at the molecular species level. Our data open a detailed and quantitative view of PC homeostasis in these cells and demonstrate differences between PCs carrying saturated or various unsaturated side chains.

## Materials and Methods

### Lipid and chemical probes

This text uses the consensus nomenclature for lipids (29) and follows published rules for alkyne lipid tracers (30). Palmitoyl-lyso-propargyl-PC (LpPC 16:0) and the ether analog 1-O-hexadecyl-2-lyso-sn-glycero-3-phosphatidylpropargylcholine (LpPC O-16:0) were synthesized as before (22, 26). The syntheses of lauroyl-lyso-propargyl-PC (LpPC 12:0), stearoyl-lyso-propargyl-PC (LpPC 18:0), palmitoleyl-lyso-propargyl-PC (LpPC 16:1 omega-7), oleoyl-lyso-propargyl-PC (LpPC 18:1 omega-9), linoleoyl-lyso-propargyl-PC (LpPC 18:2 omega-6), α-linolenoyl-lyso-propargyl-PC (LpPC 18:3 omega-3), arachidonoyl-lyso-propargyl-PC (LpPC 20:4 omega-6) and docosahexanoyl-lyso-propargyl-PC (LpPC 22:6 omega-3) from the respective *sn-1,2*-homoacyl-3-phosphatidylpropargylcholine and *sn-1,2*-homoacyl-3-phosphatidylcholine (Avanti) were performed analogously as described (22).

The synthesis of the MS-reporter azide (azido-palmitate, azPal) was described before (22). Analogously, azido-undecanoate (azUnd) was synthesized as follows. 11-Bromoundecanoic acid (Sigma, B82904) was stirred with sodium azide (Sigma, 8223350) in dimethyl sulfoxide for 24 h. Hexane/ethylacetate 3/1 was added to the reaction mix before several extractions with water. The organic phase was separated and the solvent evaporated. Pure azUnd was crystallized from hexane/ethylacetate 3/1. Azido-nonanoate (azNon) and azido-decanoate (azDec) were analogously obtained from 9-bromononanoic acid (Sigma, T333328) or 10-bromodecanoic acid (Sigma, 541397), respectively. For the synthesis of azido-pentadecanoate (azPen) 15-hydroxypentadecanoate (Sigma, 392979) was esterified in methanol. 15-Hydroxypentadecanoate methyl ester was stirred with mesyl chloride and pyridine for 3 h before drying. Upon addition of hexane/ethylacetate 1/1 and saturated NaHCO_3_ the organic phase was dried and 15-mesylpentadecanoate methyl ester was obtained, which was stirred with sodium azide in dimethyl sulfoxide for 24 h. Hexane/ethylacetate 3/1 was added to the reaction mix before several extractions with water. The organic phase was separated and the solvent evaporated. The obtained 15-azidopentadecanoate methyl ester was purified by silica column chromatography before alkaline ester hydrolyzation in tetrahydrofuran. Upon pH neutralization and addition of hexane/ethylacetate 1/1, the organic phase was dried to yield pure azPen. Azido-oleate (azOle) was purchased from Avanti (900415C).

### Cell culture and lipid labeling

The brain endothelial cell line bEND3 (31) was obtained from ATCC (CRL-2299) and maintained in DMEM medium (Gibco, 31966021) containing 10% fetal calf serum (Gibco, A5256701) and 1% penicillin/streptomycin (Gibco, 15070063). For experiments 75,000 cells/well were seeded into 12-well dishes. Propargylcholine lipids were added to the medium at concentrations of 50 μM from 5-10 mM stock solutions in 80% ethanol. Cells were then cultured for 10 min to 24 h as indicated.

### Isolation and culture of hepatocytes

Hepatocytes from eight-week-old male C57BL/6N mice were isolated by a two-step collagenase liver perfusion as described (30). All animal experiments were approved by the Institutional Animal Care and Use Committee (North Rhine Westfalia, Germany); permission LANUV NRW, 81–02.04.2021.A166. Hepatocytes 75,000 cells/well were plated onto collagen-coated 12-well dishes and incubated in DMEM medium. Three hours after plating, the media was replaced by DMEM medium containing 50 μM propargylcholine lipids, and cells were cultured for 10 min to 4 h, as indicated.

### Lipid extraction and click-reaction for TLC

The protocol followed the literature (25) with minor adaptations. Cells were washed once with ice-cold PBS (Sigma, 806552), and cellular lipids were extracted by sonication of multi-well plates in MeOH/CHCl_3_ 5/1. Extracts were retrieved and proteins precipitating from the one-phase mix were removed before a two-phase separation of lipids. The dried lipids were redissolved in 10 μl CHCl_3_. After addition of 40 μl of coumarin mix (10 μl 3-azido-7-hydroxycoumarin (2 mg/ml in acetonitrile), 250 μl of 10 mM Cu(I)BF_4_ in acetonitrile, 850 μl of EtOH) samples were incubated either at 42°C for 16 h in a heating block (regular protocol) or at −80°C for 15 min (accelerated protocol). After the click reaction, samples were separated by TLC on HPTLC plates (Merck) using two solvent mixes sequentially (solvent I: CHCl_3_/MeOH/H_2_O/acetic acid 60/40/5/1; solvent II: hexane/ethyl acetate 1/1), dried and briefly soaked in 4% N,N-diisopropylethylamine in hexane). Fluorescence images of TLC plates illuminated by a 420 nm light source were acquired with a Rolera MGI plus EMCCD camera (Decon Science Tec), equipped with a 494/20 nm emission filter, all under control of GelPro analyzer (Media Cybernetics) software.

### Lipid extraction and click-reaction for MS

Cells were washed once with warm medium, once with ice-cold PBS, and once with 155 mM ammonium acetate, taking care to remove the liquid after the last wash. The lipids were extracted by addition of 500 μl methanol:chloroform 5/1 containing 240 pmol pPC 31:1, 250 pmol LpPC 14:0, 250 pmol PE 33:1(d7), 472 pmol PC 33:1(d7), 98 pmol PS 31:1, 56 pmol PA 31:1, 51 pmol PG 28:0, 39 pmol LPA 17:0, 35 pmol LPC 17:1, 38 pmol LPE 17:1, 32 pmol Cer 17:0, 241 pmol SM 18:1(d9), 55 pmol GlcCer 12:0, 339.7 pmol TG 50:1(d4), 111 pmol CE 17:1, 64 pmol DG 31:1 and 103 pmol MG 17:1 as internal standard (22, 32). Culture dishes were sonicated in a bath sonicator for 30 s before lipid collection. After centrifugation, the supernatants were retrieved and mixed 300 μl chloroform and 700 μl of 1% acetic acid to induce phase separation. The organic phase was collected, evaporated in the speed vac (45°C, 20 min) and redissolved in 10 μl chloroform and the tubes briefly vortexed. To each tube 70 μl of click-mix was added (prepared by mixing 10 μl of 50 mM either azNon or azDec or azUnd or azPen or azPal or in ethanol with 250 μl 5 mM Cu(I)AcCN_4_BF_4_ in AcCN and 750 μl ethanol) before sonication for 5 min. Click reaction was performed by incubating either at 42°C for 16 h (regular protocol) or −80°C for 15 min (accelerated protocol). 100 μl CHCl_3_ was added, samples pooled into multiplexes, and a twofold excess of water was added. Samples were briefly shaken and centrifuged for 5 min at 20,000*g*. The upper phase was removed and the lower phase dried in a speed-vac as above. 1,200 μl of spray buffer (2-propanol/methanol/water 8/5/1 + 10 mM ammonium acetate) was added, the tubes were sonicated for 5 min and stored at −20°C.

### MS analysis

The tubes were sonicated for 5 min and the dissolved lipids were analyzed. Mass spectra were recorded on a Thermo Q-Exactive Plus spectrometer equipped with a standard HESI ion source using direct injection from a Thermo Dionex AS-AP autosampler driven by an AXP-MS pump under the control of Xcalibur software. MS1 spectra (resolution 280,000) were recorded in 100 *m/z* windows from 250–1,200 *m/z* (positive mode) and 950–1,300 *m/z* (negative mode) followed by recording MS/MS spectra (resolution 280,000) by data-independent acquisition in 1 *m/z* windows from 200–1,200 *m/z* (positive mode) and 950–1,300 *m/z* (negative mode).

### MS data analysis

Raw files were converted to mzml files using MSConvert and analyzed using LipidXplorer (33). For identification and quantification of labeled alkyne lipids, molecular fragment query language (mfql) files were written that identify the species by the presence of a peak corresponding to the expected masses of the labeled lipid class combined with the characteristic neutral loss (22). Labeled pPC was quantified using the pPC 31:1 internal standard. MS2 signals of the click-reacted lipid (PR2), its diagnostic fragmentation peak upon neutral loss, and those of the respective FAs were recorded. Endogenous lipids were quantified using the respective internal standard.

## Results

### Exogenous lyso-lipid precursors yield labeled choline phospholipids upon cellular acylation

**Fig. 1:**
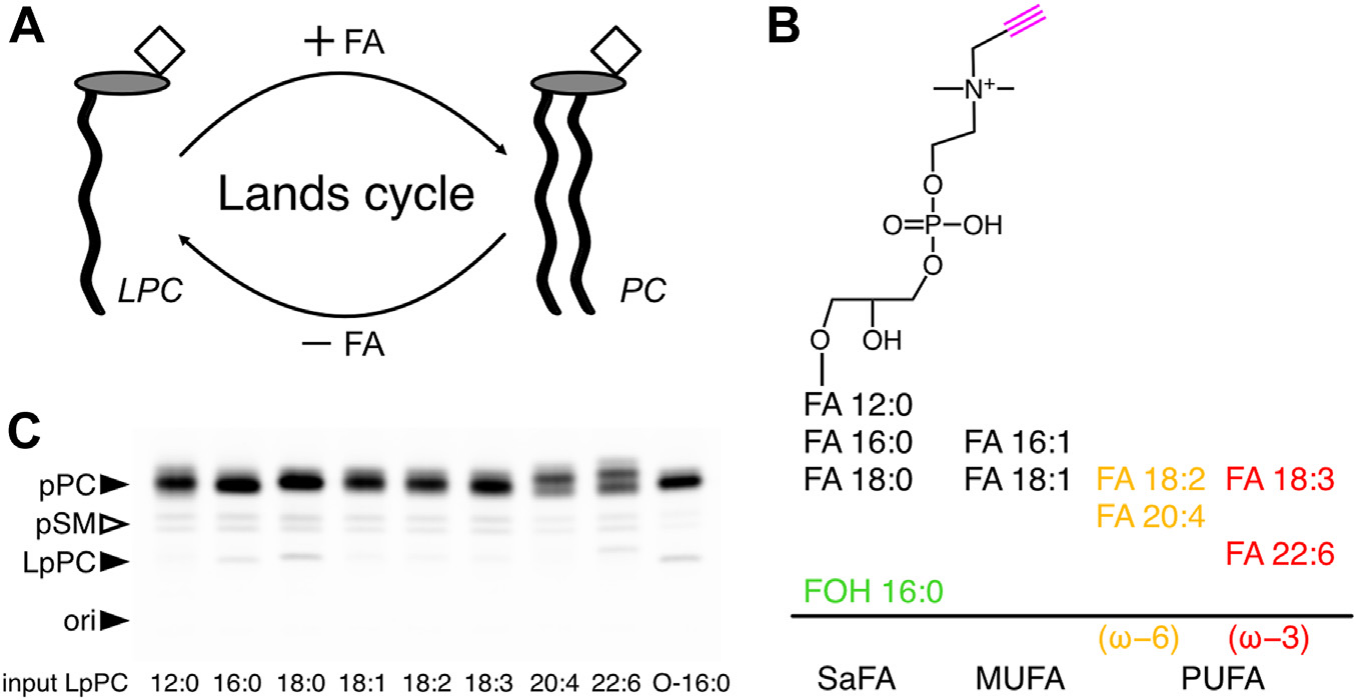
Tracing of glycerophosphocholine metabolism. (A) Cellular acylation of lyso-phosphatidylcholine (LPC) by fatty acids (FA, or other acyl donors) yields phosphatidylcholine (PC) and upon de-acylation LPC is re-produced. This Lands cycle allows for side-chain exchange. (B) A library of synthetic head group-labeled (magenta) alkyne tracers featuring saturated (SaFA), mono-unsaturated (MUFA), or poly-unsaturated (PUFA) fatty acid side chains at the *sn-1* position were synthesized. The N-propargyl-*sn2*-lysophosphatidylcholine (LpPC) tracer family includes members with ω−6 (orange) and ω−3 (red) PUFA side chains. Also an ether derivative (LpPCO) containing an *sn1*-O-alkyl side chain (green) was synthesized. (C) bEND3 cells were incubated for 24 h with 50 μM LpPC containing the indicated side chain. Total lipids from cells were isolated, click-reacted and analyzed by TLC. Fluorescent bands were identified by co-migrating standards (closed arrowheads). Cellular pPC metabolites were detected as multiple bands. The double band migrating between LpPC and pPC putatively represents pSM (open arrowhead). ori, origin of application.

Cellular acylation of LPC via the Lands cycle generates PC (Fig. 1A). To investigate PC metabolism and the effect of various side chains we chose a strategy based on lyso-phosphatidylpropargylcholine (LpPC) precursors. A library of these head group-labeled alkyne analogs differing in the length and double bond number of the *sn-1* side chain was synthesized (Fig. 1B). An ether lipid precursor, LpPC O-16:0 with the corresponding fatty alcohol at the *sn-1* position complemented the library (22). The brain endothelial cell line bEND3 was incubated with 50 μM of each tracer for 24 h and the metabolic fate of the precursors were analyzed by TLC (Fig. 1C). Upon uptake the various LpPCs or the LpPC O-16:0 underwent cellular acylation to yield phosphatidylpropargylcholine (pPC) or its ether companion pPC O, respectively. The pPC pool accounted for the majority of labeled metabolites but small amounts of putative propargylsphingomyelin (pSM) likely produced by head group exchange were also detectable. The latter formed a double band likely reflecting the major SM species with 16 or 24 carbon chains. Similarly, the pPC metabolite formed a pool of different molecular species as indicated by its non-uniform band that fragmented into several bands nearly comigrating. Albeit of equal overall intensity, the sub-patterning of the pPC band differed for the precursors with various side chains. Already upon 30 min incubation (supplemental Fig. S1A–I), the first time point investigated, the sub-patterning within the pPC band resembled that of all later time points (1–8 h) as well as that after 24 h (Fig. 1C). However, due to limited band separation and the lack of suitable comigrating standards no further details on molecular species could be obtained. The cells preserved little to no LpPC educt. In the media the amount of LpPC educt remained constant over all time points and no secreted pPC metabolites could be detected (supplemental Fig. S1A–I).

### A novel protocol for multiplexed mass spectrometry analysis

For assessment of the added side chain preferred in the acylation and for pPC molecular species identification a mass spectrometry (MS) approach was chosen. We first adapted our established analysis to include sample multiplexing for a higher sample throughput. Based on the original azide reporter, azido-palmitate (azPal) for propargylcholine-containing phospholipids (22), a family of five related azide reporters (supplemental Fig. S2A) was synthesized, including azido-nonanoate (azNon), -decanoate (azDec), -undecanoate (azUnd), -pentanoate (azPen) and -oleate (azOle). Sharing the relevant functionalities i.e. the carbonyl and azido groups, they mainly differed in the length of the carbon chain. Upon click reaction to an analyte (supplemental Fig. S2B), the tagged lipids carry a permanent negative charge (supplemental Fig. S2C). The additionally conveyed mass shift differs for each reporter. In a multiplexing experiment, six individual samples are click-reacted to one reporter each and pooled. The conveyed mass shifts allow for direct identification at the MS1 level (supplemental Fig. S2D). In MS2, the reacted molecule shows stereotypical fragmentation by undergoing a neutral loss (NL) characteristic for each reporter (supplemental Fig. S2E). As the analysis is performed in the negative mode, it also identifies both FA side chains (supplemental Fig. S2F).

To demonstrate the applicability of the new multiplexing method a proof-of-concept experiment was performed. Four tracers were chosen, LpPC 16:0, 18:2, 22:6, and the ether LpPC O-16:0. Endothelial cells were incubated with 50 μM of tracer for 24 h (supplemental Fig. S3A). Cellular lipids were extracted in the presence of internal standards and the extracts were divided into six aliquots. Each aliquot was click-reacted to one of the six azido reporters before pooling for hexuplet formation and MS analysis. Parallel samples where each azido reporter was analyzed individually, demonstrated that no analytical crosstalk between lipidomes click-reacted to the six different reporters occurred (supplemental Fig. S3B). Using LpPC 16:0 tracer and hexuplet analysis (supplemental Fig. S3C) MS revealed the dominant labeling of pPC 34:1 (43 mol%), nearly doubling the relative content of PC 34:1 of untreated controls (23 mol%). This indicated a metabolic acylation of the input tracer with the abundant oleate. Other labeled pPC species were detectable and amongst them, pPC 32:1 and 36:1 also showed increased relative amounts compared to controls. Examining the data on the most abundant species pPC 34:1 from the five biological replicates as analyzed by the six azido reporters in a total of 30 determinations, an overall variability of 11% over all measurements was achieved, with the biological variability accounting for 5% therein.

When cells were incubated with the unsaturated LpPC 18:2 or LpPC 22:6 different labeling profiles emerged. For LpPC 18:2 (supplemental Fig. S3D) an increase in the relative amounts of labeled pPC 34:2, 36:2, and 36:3 compared to controls indicated a preferential tracer acylation with palmitate, stearate, and oleate, respectively. The labeling of PC 34:1, generally the most frequent species in control samples, was found markedly reduced. The fact that labeled saturated and mono-unsaturated pPC 32:0, 32:1, 34:1, and 36:1 originated from poly-unsaturated LpPC 18:2 pointed to substantial lipid remodeling. Labeling by LpPC 22:6 (supplemental Fig. S3E) yielded elevated relative amounts of poly-unsaturated pPC 38:6 and 40:6, indicative of palmitate and stearate serving as acylation partners. Abundant labeling of species with less than six double bonds supported the notion of extensive lipid remodeling.

The ether lipid precursor LpPC O-16:0 gave rise to high amounts of ether pPC O-34:1 (39 mol%) and pPC O-32:1 (21 mol%), an increase compared to the respective unlabeled species in controls (30 mol% PC O-34:1; 12 mol% PC O-32:1; supplemental Fig. S3F). About 13% of all labels was conveyed from the ether to the non-ether PC pool by head group transfer within 24 h of the experiment.

As these proof-of-concept experiments demonstrate the applicability of the new multiplexing method, the collective data obtained here (supplemental Fig. S3C–F) also indicate the large extent of lipid remodeling in the PC pool over 24 h.

### Kinetic analysis of PC metabolism in endothelial cells

**Fig. 2:**
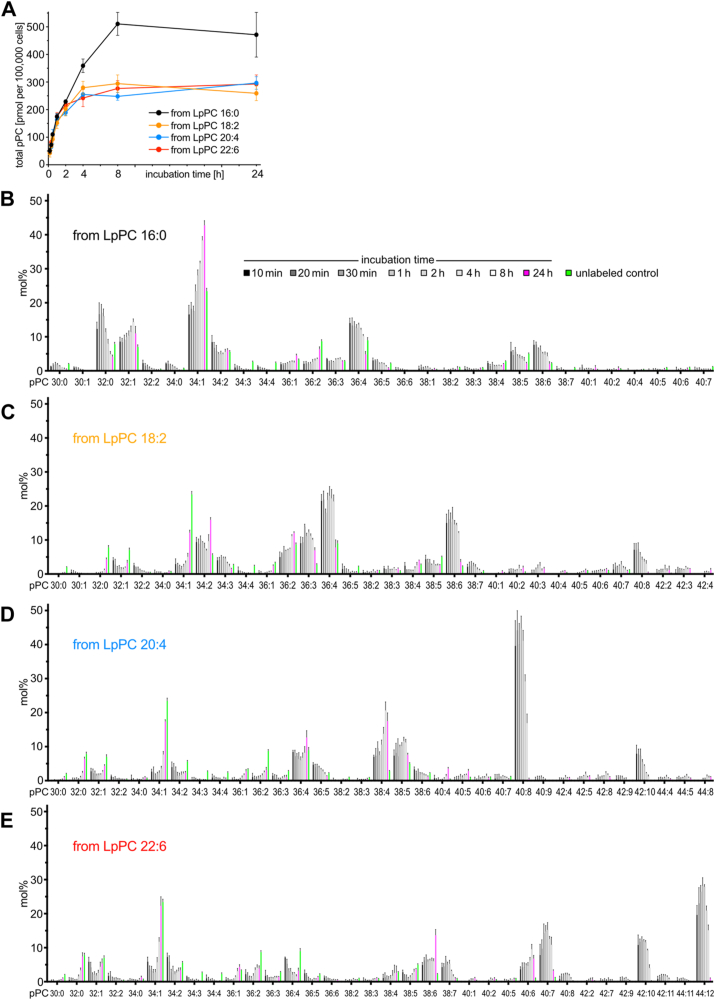
MS analysis of pPC metabolism in bEND3 cells over time. Cells were incubated with one of four LpPC tracers (50 μM) for various times. Total cellular lipids and internal standards were co-isolated and click-reacted before multiplexed MS analysis. All labeled pPC species were quantified (see supplemental Tables S1–S4) and the total cellular pPC content per 100,000 cells is plotted over time (A). Error bars smaller than symbol size are omitted. Cells incubated with (B) LpPC 16:0, (C) LpPC 18:2, (D) LpPC 20:4, or (E) LpPC 22:6 showed distinct distributions of labeled pPC species at each time point. Upon 24 h (magenta bars) of constant tracer supply and ongoing cellular lipid remodeling the labeled lipidome differed substantially from earlier time points. The species distribution of unlabeled control samples (green bars) is also shown. Labeled pPC species are presented as the molar fraction (mol%) of all labeled pPC species. N = 3.

Next, a time course feeding experiment with shorter incubation times and MS analysis was performed using endothelial cells (Fig. 2). Four LpPC tracers with saturated (16:0) or poly-unsaturated omega-6 (18:2; 20:4) or omega-3 (22:6) side chains and eight time points were chosen to assess whether cellular acylation temporarily accumulates certain pPC molecular species that undergo subsequent remodeling before converging to a steady-state-distribution of pPCs. Using our new approach, 96 individual samples were analyzed as 16 multiplex samples by MS. All labeled pPC species derived from each precursor were discretely identified and quantified at each time point (supplemental Tables S1–S4). The total amount of labeled pPC increased over time displaying the strongest gain within the first hour of incubation and a flattening curve after 4–24 h (Fig. 2A). While at early time points (up to 2 h) all four tracers gave similar amounts of labeled pPC metabolites, the saturated LpPC 16:0 yielded substantially more labeled pPC after 4–24 h incubation time than the poly-unsaturated tracers. Amongst the latter, no difference between the omega-6 and omega-3 side chains was observed.

For each tracer, a main set of labeled pPC species was detectable as early as 10 min post incubation start (supplemental Tables S1–S4). Importantly, the single most abundant species at this earliest time point varied for each precursor. While pPC 34:1 was predominant for LpPC 16:0, the poly-unsaturated LpPC 18:2, 20:4 or 22:6 tracers yielded prevalently pPC 36:4 or 40:8 or 44:12, respectively. The latter poly-unsaturated pPC species themselves were further characterized as being mostly pPC 18:2/18:2, 20:4/20:4, and 22:6/22:6, respectively, carrying two identical FAs each. This prevalence of pPC 36:4 or 40:8 or 44:12 persisted for 4–8 h of incubation or, in the case of pPC 34:1 from LpPC 16:0, for the whole experimental time of 24 h.

When the molar fraction of each species was plotted over the course of the experiment (Fig. 2B–E) the time-dependent shifts in the species distribution became apparent. While for LpPC 16:0 (Fig. 2B) the early predominance of pPC 34:1 was only modest, the relative content of this metabolite strongly increased during the experiment reaching finally 43 mol%. Conversely, the initially abundant pPC 36:4 and 38:6 declined over time and so did pPC 32:0 after peaking between 20-60 min of incubation. For LpPC 18:2 (Fig. 2C) the early prevalence of pPC 36:4 and 38:6 faded after 8 h when pPC 34:1, 34:2 and 36:2 became most abundant. Other early species like pPC 38:7 and 40:8 had largely disappeared by 24 h. Incubation with LpPC 20:4 (Fig. 2D) yielded the strongly dominant pPC 40:8 that accounted for 40 mol% of all labeled PC already at the earliest time point investigated. This large fraction remained stable for 2 h, before gradually dropping to less than 1 mol% by the end of the experiment. The early species pPC 42:10 had also vanished by then. Conversely, the relative abundance of other early species like pPC 36:4 and 38:4 increased with time as did the fraction of pPC 34:1. Upon incubation with LpPC 22:6 (Fig. 2E) the pPC 44:12 remained the predominant metabolite for all time points but the last. Peaking at 28 mol% after 1 h this species had largely disappeared by 24 h and pPC 34:1 became the prevalent species instead. Also, pPC 38:6 and 40:6 increased in relative abundance whereas the fraction of the early species pPC 40:7 substantially decreased.

The unlabeled lipidome was also investigated (supplemental Fig. S4A–L). Here, the analysis of the pooled hexuplet samples focused on abundant lipid classes and species, that likely contain the respective side chain of the input tracer (FA 16:0, 18:2, 20:4, or 22:6). The samples of LpPC 16:0 or 20:4 incubations were pooled as were those of the LpPC 18:2 or 22:6 tracers. The relative amount of lipids (mol%) was followed over time to assess the dispersion of the input side chain into the unlabeled lipidome e.g. upon tracer hydrolysis or by side chain remodeling. The pool of unlabeled PC decreased over time (supplemental Fig. S4A, G) as the amount of labeled pPC increased (Fig. 2A) an effect seen before (22). Performed as positive-mode MS analysis no molecular species resolution was obtained for PC, PE, and PS species (supplemental Fig. S4B–D, H–J). For LPC, LPE, CE, and TG one acyl chain was identified (supplemental Fig. S4E, F, K, L). Together these data suggested that over time the poly-unsaturated side chains of LpPC 20:4 and 22:6 accumulated more strongly in the unlabeled lipidome than the side chains of LpPC 16:0 and 18:2.

Integrating all data from the endothelial cells, the early-on occurrence of large amounts of labeled PC carrying two copies of the input side chain was intriguing but was only prominent for the poly-unsaturated LpPC tracers. LpPC 18:2 or 20:4 or 22:6 yielded pPC 18:2/18:2 or 20:4/20:4 or 22:6/22:6, respectively. The existence of each of these species was largely confined to samples employing the respective precursor and rather lacking in other samples, that were labeled by different LpPCs or in unlabeled controls. It indicated a preferential use of the tracer’s side chain for acylating a second tracer molecule. As this phenomenon was observed primarily for the poly-unsaturated educts it also indicated a difference in metabolic handling of the saturated versus poly-unsaturated LpPC precursors.

### Kinetic analysis of PC metabolism in hepatocytes

Next, a similar time course experiment using a different cell type, primary hepatocytes, was performed and the labeled pPC metabolites of the four tracers were quantified (supplemental Tables S5–S8). The total amount of labeled pPC increased over time (supplemental Fig. S5A). Already at 10 min pPC 36:4 was found to be the main labeled pPC species for all tracers (supplemental Fig. S5B–E). Throughout the experiment, pPC 36:4 remained dominant for LpPC 16:0 and 20:4 (supplemental Fig. S5B, D) or remained a major species for LpPC 18:2 and 22:6 incubation (supplemental Fig. S5C, E). A prevalence for pPCs carrying two FA copies of the input side chain, i.e. pPC 16:0/16:0, 18:2/18:2, 20:4/20:4, and 22:6/22:6, was generally not detected although a temporary bias for pPC 20:4/20:4 from LpPC 20:4 was seen between time points 20 min and 2 h (supplemental Tables S5–S8). Remarkably, in hepatocytes and for all four tracers the labeling was directed toward poly-unsaturated pPCs, likely produced with arachidonate serving as acylation partner. Overall, the labeled pPC pool appeared narrow and e.g. pPC 34:1 or other species contained only minor amounts of the label. The dispersion of the input tracers side chain (FA 16:0, 18:2, 20:4, or 22:6) into the unlabeled lipidome appeared similar for all four tracers (supplemental Fig. S6A–P).

Comparing the data of hepatocytes and endothelial cells some disparities became apparent indicating cell-specific differences in metabolic handling during the acute phase of lipid influx. The endothelial cells generated substantial amounts of pPC 18:2/18:2 or 20:4/20:4 or 22:6/22:6 from LpPC 18:2 or 20:4 or 22:6, respectively, but in hepatocytes, these unusual metabolites mostly were of low abundance. Intriguingly, in endothelial cells, these symmetric pPC species occurred early on but were only prominent for the poly-unsaturated LpPC tracers. In hepatocytes, no such prevalence was observed as only pPC 20:4/20:4 reached a notable concentration.

### Kinetic analysis of PC remodeling in endothelial cells

**Fig. 3:**
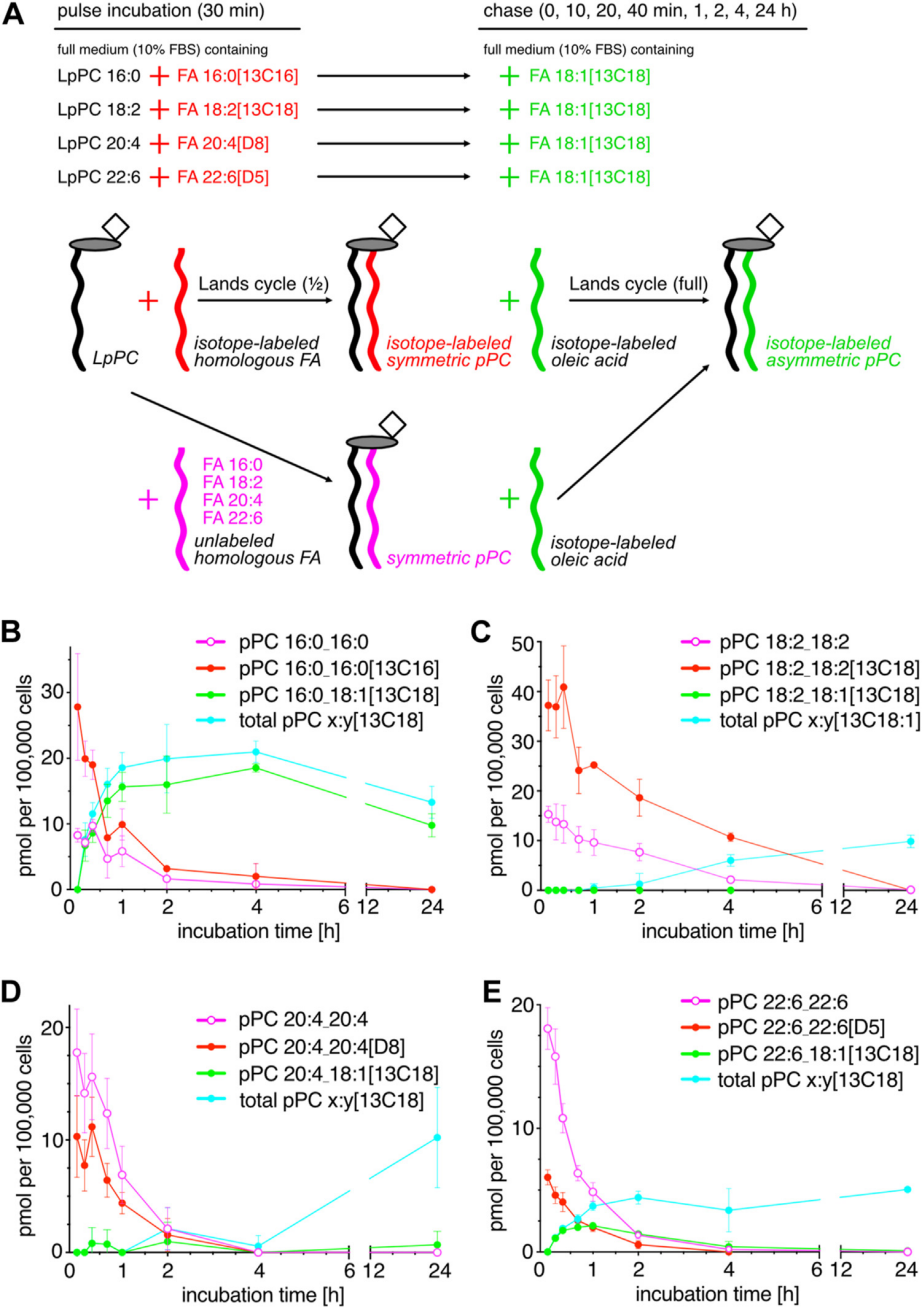
Analysis of LPC acylation and PC side chain remodeling in bEND cells. (A) Experimental scheme: For 30 min bEND3 cells were pulse-incubated with 50 μM LpPC containing the indicated side chain and 50 μM of the homologous isotope-labeled FA (red) to generated isotope-labeled symmetric pPC metabolites. Symmetric pPC lacking an isotope label was produced if the unlabeled homologous FA (magenta) served as acylation partner. Cells were chased in the presence of isotope-labeled oleate (green) for the indicated time during which side chain replacement yielded labeled asymmetric pPC. (B–E) The amount of selected pPC species is presented over time as detected by MS in lipid extracts from cells incubated with (B) LpPC 16:0, (C) LpPC 18:2, (D) LpPC 20:4 and (E) LpPC 22:6. The isotope-labeled symmetric pPC (red) and the symmetric pPC lacking an isotope label (magenta) were produced first during the pulse. Generated upon side chain remodeling, the pPC species carrying the side chain of the respective input LpPC as well as the 13C-oleate added during chase is highlighted in green. The sum of all pPC species labeled by 13C-oleate during the chase is shown in cyan. N = 3.

The striking generation of symmetrical pPC with identical side chains in endothelial cells poses the question of the origin of the side chain used in the acylation reaction. Furthermore, the existence of these pPC species offers an attractive test system to investigate the kinetics of side chain remodeling. To study both topics in more detail a series of pulse-chase experiments was performed (Fig. 3A). For that, endothelial cells were pulse-incubated with LpPC and its homologous isotope-labeled free FA. During the subsequent chase period, both pulse tracers were absent, but isotope-labeled oleic acid was provided. Its incorporation into pPC by side chain remodeling during the chase phase could be followed over time while the amount of the symmetrical pPC species simultaneously faded.

When pulse-incubated with the tracer tandem of saturated 16:0 chains, the symmetric pPC was generated using isotope-labeled or unlabeled side chain at a 3:1 ratio (Fig. 3B, compare the origin of the red and magenta curves). For the tandems of poly-unsaturated 18:2, 20:4 or 22:6 chains this ratio was about 2:1, 2:3, and 1:3, respectively (Fig. 3C–E, compare origin of the red and magenta curves). As this ratio shifts with increasing chain length and double bond count a dissimilar metabolic handling of these LpPC is indicated. These differences may originate from different acylases handling the various LpPCs at distinct cellular loci. However, as analyzed by fluorescence microscopy no obvious distinction in subcellular localization of the different labeled lipids was noticed after 10 min incubation (supplemental Fig. S7A–D) or 30 min. For all four tracers a widespread staining of numerous cellular membranes in the perinuclear region and of the endoplasmic reticulum, mitochondria, and plasma membrane, all identified by morphology, was observed. No accumulation at specific cell structures for any of the tracers was detected.

**Fig. 4:**
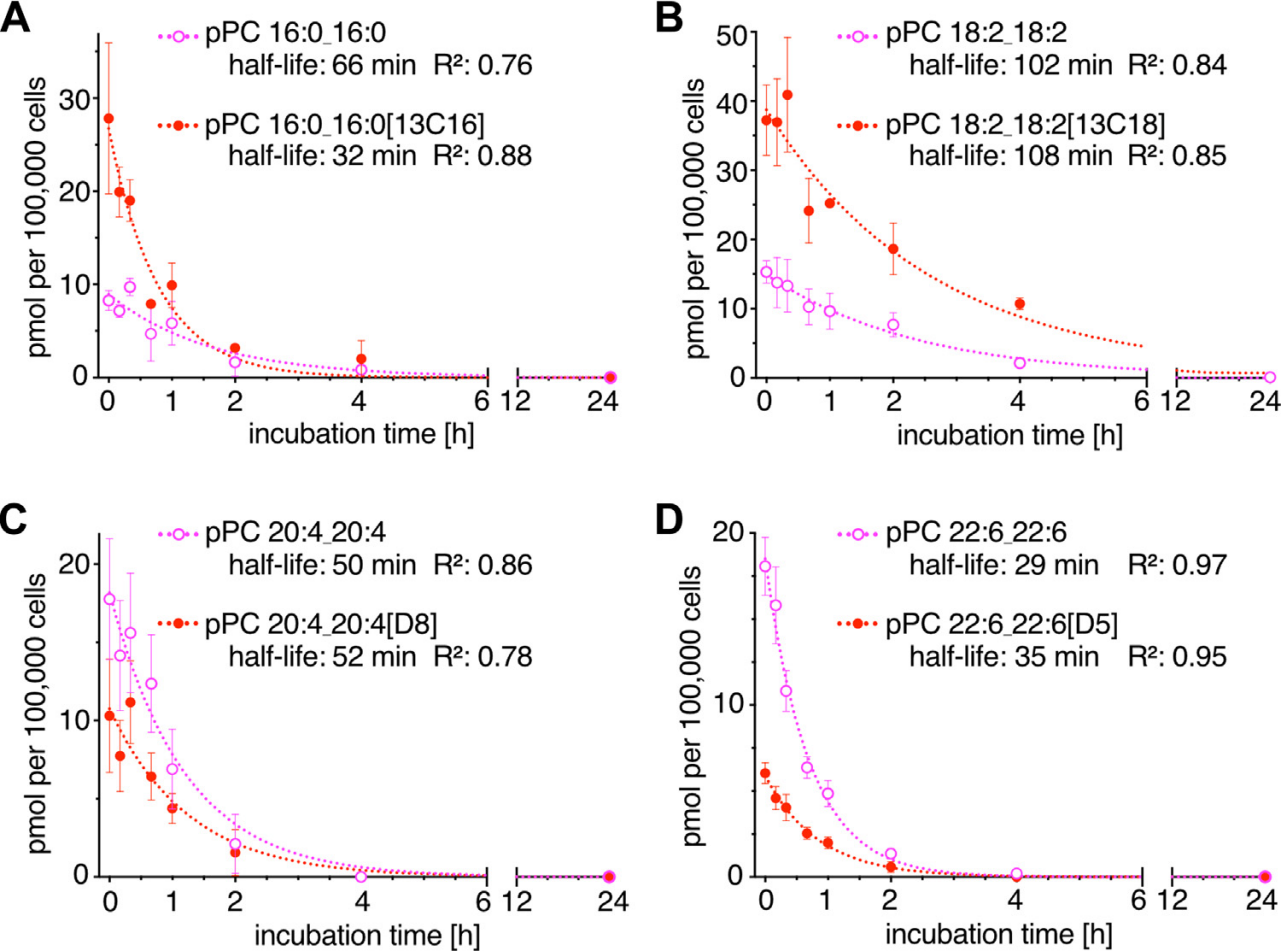
Kinetic analysis of the decomposition of symmetric pPC species. Symmetric pPC species were generated from (A) LpPC 16:0, (B) LpPC 18:2, (C) LpPC 20:4, and (D) LpPC 22:6 were followed over time as displayed in Figure 3B–E. The data were fitted by nonlinear regression to a first-order (one-phase) exponential decay curve. The half-lives of the symmetric pPC with an isotope-labeled (red) or two unlabeled side chains (magenta) were deduced. Adjusted *R*^2^ values are given.

When analyzing the decay of the symmetric pPC generated during the pulse it is important to note that every modification at either *sn-1*, *sn-2* or *sn-3* position would result in a decrease of the signal. This includes de-acylation or head group modifications as well as side chain oxidations or alterations of chain length and saturation. For simplification, the analysis may ignore all latter processes and only considers the de-acylation reaction. If, furthermore, one assumes that a given symmetric pPC species is either exclusively subjected to *sn-1* or *sn-2* de-acylation and no re-synthesis of the very same symmetric pPC by the Lands cycle occurs, the decay data can be fitted to a first-order exponential decay curve (Fig. 4A–D). The deduced half-lives for the poly-unsaturated symmetric pPCs decreased with increasing chain length and double bond count and were similar for pPCs containing an isotope-labeled or two unlabeled side chains (Fig. 4B–D). In contrast, the saturated pPC 16:0_16:0 showed a twofold longer half-life than pPC 16:0_16:0[13C16] (Fig. 4A). After 4 h of chase most symmetric pPCs had virtually disappeared with the notable exception of pPC 36:4 generated from LpPC 18:2 (Figs. 3 and 4). In the course of the 24 h chase also the total amount of all other pPC species originally present after the pulse incubation was reduced (supplemental Tables S9–S12).

Side chain remodeling where isotope-labelled oleate replaced one FA in the symmetric pPC during chase was demonstrated in three experimental series as pPC 16:0_18:1[13C18], pPC 20:4_18:1[13C18] and pPC 22:6_18:1[13C18] (Fig. 3B–E, respectively, green curves) became detectable. During the chase, these molecular species increased before fading again, and only the former reached considerable amounts peaking after 4 h. Apart from the symmetric pPC also other pPC species were targeted by the isotope-labelled oleate during remodeling (Fig. 3B–E, blue curves), but only minor quantities in a relatively narrow distribution were detected (supplemental Tables S9–S12).

## Discussion

Endothelial cells are exposed to fluctuating levels of lipids carried in the blood. Upon uptake, these lipids can serve as building blocks for lipid synthesis in the endothelial or adjacent parenchymal cells. In the case of LPC a cellular acylation yields PC, but the acylation competes with other metabolic reactions, many of which hydrolyze and degrade the LPC. Using an endothelial cell line from murine brain and head group-labeled alkyne tracers of LPC developed here and before (22, 26) we followed PC metabolism by TLC and MS. Focusing on LpPC tracers with a saturated or an omega-6 or omega-3 poly-unsaturated side chain we noted the symmetric pPCs that formed apparently by addition of a second copy of the tracers side chain. Prominently, the poly-unsaturated LpPC 18:2, 20:4 or 22:6 yielded symmetric pPC 18:2/18:2, 20:4/20:4, 22:6/22:6, respectively, but the symmetric pPC 16:0/16:0 was produced from LpPC 16:0 only at a lower frequency.

The generation of symmetric PCs was reported before: When primary hepatocytes were incubated with high loads of free unsaturated FA (1–9 mM FA 16:1, 18:1/2/3, 20:4/5, 22:6) symmetric PCs (and DGs and PEs) were formed (34, 35, 36). In contrast, from 0.1-0.9 mM of saturated FA 18:0 no PC 18:0/18:0 was produced (37). Alike, no PC 16:0/16:0 resulted from 0.1 mM FA 16:0, although at higher concentrations (0.4–0.9 mM (37)) or in other studies (38) this PC species was found. In human neutrophils, PC 20:4/20:4 was the major PC generated from high loads of FA 20:4 (39). It is important to emphasize, that free FA can enter PC synthesis either by the Kennedy pathway or Lands cycling, and both processes operate simultaneously. Parallel incubation of hepatocytes with [U-14C]glycerol and 0.3 mM unlabeled FA 16:0, 18:1, 20:4 for 20–60 min yielded the highest amount of label in symmetric PC 32:0, 36:2, and PC 40:8, respectively, despite the fact that these PC species only accounted for 0.8, 1.0 and 1.4 mol% of all PC, respectively (40). The high specific radioactivity in these minor species indicated that they were synthesized de novo by the Kennedy pathway (40).

The current study uses labeled LpPC rather than FA for tracing. The LpPC as such is likely subjected to the Lands cycle yielding labeled pPC, while a fraction may also be hydrolyzed. Thus, its liberated side chain would follow the metabolic fate of a FA and could re-enter the labeled lipid pool by serving as acyl donor in the Lands cycle or Kennedy pathway. Once liberated, however, the FA would compete with all other FAs of the cellular pool. The fact that we find symmetric pPCs being produced may be explained by *i)* a bias for symmetric products by the LPLATs, *ii)* a trans-acylation process between two LpPC molecules, *iii)* a large endogenous pool of the respective FA, *iv)* a hydrolyzation of the LpPC tracer liberating the FA, or *v)* a local confinement of reactants and enzymes.

The expression pattern of LPLATs in the brain endothelial cell line bEND3 has not been determined, but seven of 14 LPLATs are expressed in the brain and at least 4 thereof possess LPCAT activity (7). However, to our knowledge, none of these were reported to show *i)* a bias for symmetric products or *ii)* evidence for a trans-acylation activity. Considering *iii)* the cellular FA composition it is well established that the FA profile of cultured cells is unlike that of natural cells with twice the mono-unsaturated and half the poly-unsaturated relative levels (41). This is a consequence of the cultivation with fetal bovine serum containing about 190 μM of total FA with only 4 μM PUFA (42). Hence, many cultured cells on average contain 26% palmitate, 15% stearate, 26% oleate, 3% linoleate, 5% arachidonate, 0.5% linolenate and 2% docosahexanoate (41) and such profile would favor an acylation by FA 16:0 and 18:1. Nonetheless, poly-unsaturated LpPC dominantly yielded symmetric pPC in endothelial cells and this dominance was established early-on and persisted for extended time periods. In the endogenous pool of unlabeled lipids the species putatively containing FA 20:4 or 22:6 increased over time, whereas those presumably holding FA 16:0 or 18:2 showed no or only a minor relative increase. Together, these findings support the idea of a *iv)* considerable hydrolyzation of the poly-unsaturated LpPCs, especially LpPC 20:4 and 22:6. As we have no quantitative data on the hydrolyzed fraction, however, it must be taken into account that a relatively small amount of liberated PUFA meets a scarce endogenous pool and a metabolic machinery that favors PUFAs in acylation reactions. This would strengthen the impact of tracer hydrolyzation and could account for the generation of the symmetric pPC species. Alternatively, *v)* a locally confined hydrolyzation and acylation sequence may promote symmetric pPC production. Judged by our microscopy study, though, all labeled lipids displayed a similarly wide distribution over all major cellular membrane systems and no distinct accumulation. Hence, albeit not ruling out this possibility no local confinement of reaction partners or activities that would exclude a wider cellular pool is indicated.

The hydrolyzation of poly-unsaturated LpPCs and the formation of symmetric pPCs containing the liberated FA were prominent in the brain endothelial cells, but not in hepatocytes. Hepatocytes that do form symmetric PCs when incubated with unsaturated FAs (34, 35, 36, 37) mostly generate asymmetric pPC species when exposed to unsaturated (or saturated) LpPCs. Arachidonate served here as a main acylation partner for all LpPC tracers, which limited the formation of symmetric pPCs except in the case of LpPC 20:4 incubation, where symmetric pPC 20:4/20:4 was of notable abundance.

Our findings on the metabolic fate of the LpPC 16:0 precursor in endothelial cells support the notion, that these cells handle saturated LPCs differently with less tracer hydrolyzation occurring. Contrary to the poly-unsaturated precursors, the saturated LpPC 16:0 yielded primarily the unsymmetric pPC 34:1. As oleate served as the main acylation partner, a major extent of precursor hydrolysis adding a big surplus of palmitate to the pool appears unlikely. However, the cellular palmitate levels are large, and the detection of tracer could be obscured by the abundance of the endogenous FA. Support for a lesser rate of saturated versus poly-unsaturated LPC hydrolyzation can be drawn from the unequal amounts of total pPC we detected for the various tracers. As hydrolyzation not only releases the FA to promote symmetric pPC synthesis but also the head group, the propargyl-choline moiety thus liberated may exit the lipidome altogether. The concentration of water-soluble metabolites was not monitored, but the loss of the head group would result in less labeled pPC. Therefore, the finding that LpPC 16:0 yielded about twice as much total pPC as the poly-unsaturated tracers may indicate that the hydrolyzation rate of the saturated is only half of that of the poly-unsaturated LpPCs.

When comparing the pPC species generated from the different tracers an additional layer of complexity must be considered as the tolerance for saturated or unsaturated side chains at the *sn-1* or *sn-2* position differs. In general, saturated FAs are found at the *sn-1* position, whereas (poly)-unsaturated FAs are commonly found at the *sn-2* position (4). While such stereotypic positioning may be bypassed during acute handling of larger amounts of incoming lipid it certainly drives side chain remodeling proceeding thereafter. Our data from endothelial cells with continuous exposure to the tracer shows a decline of the unusual symmetric species over time while the pool of labeled pPCs converged towards a side chain distribution matching the general scheme. This occurs despite a constant and sufficient supply of tracer well in excess of its unlabeled LPC species in the media that therefore is unlikely to compete and dilute the specific activity of the probe. The convergence coincides with the plateau formation of the total pPC concentration. The reason for the saturation effect is unclear but may related to limitations in tracer uptake or shifts in metabolic processing with an increased tracer hydrolysis fueling ß-oxidation of the liberated FA.

Side chain remodeling is an endless process. The occurrence of symmetric pPC species offered an appealing possibility to study this process in brain endothelial cells. Our simplified kinetic analysis of the decay of various symmetric pPCs provided half-lives between 0.5-2 h. These values are in line with parameters determined in primary rat hepatocytes before, where 68% of PC 16:0/16:0 and 44% of PC 18:2/18:2 were degraded within 2 h (35) and 90% of PC 20:4/20:4 within 3 h (36). There, it was concluded that PC 16:0/16:0 was subject to PLA_2_ degradation and PC 18:2/18:2 to PLA_1_ editing (35). Such biased processing would allow the most direct conversion of symmetrical PCs into canonical species with saturated and unsaturated FAs at the *sn-1* and *sn-2* positions, respectively. If all poly-unsaturated pPCs are primarily handled by PLA_1_ and we find their half-lives shortening with increased chain length and double bond count, a higher affinity of PLA_1_ for highly unsaturated PCs or a lower tolerance for these symmetric species in cellular membranes may be indicated. One possible driver for their degradation could be their biophysical properties impacting membrane permeability and lipid packing (43). The relatively long half-life of symmetric pPC 36:4 could argue for a high tolerance of species featuring four double bonds, even as the pPC 18:2/18:2 holds a poly-unsaturated linoleate chain at the unfavorable *sn-1* position.

The kinetic data for the saturated symmetric pPC 32:0 distinctly differed from that of the poly-unsaturated symmetric pPCs. Here, the isotope-labeled pPC 16:0_16:0[13C16] was profoundly more short-lived (32 min) than its sibling pPC 16:0_16:0 lacking an isotope-labeled side chain (66 min). The reason is unclear but could be explained if assuming distinct local pools of lipid being processed by different PLA_2_-activities. However, such a more complex model cannot adequately be fitted to a first-order exponential decay curve. Alike, our curve fitting did not account for additional, co-occurring reactions acting on the symmetric pPC molecules such as head group exchange or side chain modifications. Nonetheless, the applied first-order exponential decay curve delivered useful fits with arguably good quality measures. The strength and value of the deduced kinetic parameters originate from the fact that they were determined in living cells under conditions mimicking the physiological lipid load. The estimated half-life for the saturated symmetric pPC 32:0 and the poly-unsaturated pPC 44:12 was similar and as short as 0.5 h. If assuming the former is processed by PLA_2_, the latter by PLA_1,_ and re-acylation occurs at a comparable rate to the de-acylation, an overall capacity for side chain remodeling by the Lands cycle can be estimated: Our data indicate that endothelial cells can cycle >99% of all their PC molecules within 3.5 h.

However, this great capacity figure is unlikely to represent the actual turnover rate of the pool. Other symmetric pPCs showed longer half-lives illustrating a non-stochastic selection of molecules destined for metabolic conversion. Apparently, certain lipid species experience a higher pressure for adapting their ill-suited properties to fit the cellular needs or tolerance criteria. Some species are turned over at markedly lower rates, a measure also conserving energy as cycling of each PC molecule consumes two ATP. Nonetheless, it is important to note that PC cycling is generally necessary to change the PC side chain composition. In the absence of Lands cycling, both FAs would remain static after initial PC assembly, because most FA modifications, e.g. elongation and desaturation, act on FA-CoAs as substrates. The same logic also applies to the clearance of damaged FAs, for example by peroxidation, from the PC pool. In a static pool damaged material would accumulate; Lands cycling gives access to damaged FAs for subsequent degradation.

In summary, our experiments demonstrate the analytical power of the combination alkyne lipids and MS detection to trace PC metabolism at the molecular species level. The time resolved pPC profiles and kinetic parameters determined in living endothelial cells demonstrate the plasticity of their PC pool, a property supporting the acute handling of lipid influx. The results also demonstrate the robustness of lipid homeostasis as endothelial cells adapt the PC pool by side chain remodeling to match their needs. We estimate that endothelial cells possess the theoretical capacity to remodel up to 99% of their PC pool within 3.5 h using the Lands cycle. However, PC species are not subjected stochastically to this remodeling pathway as different species show different decay kinetics. Our findings emphasize the essential function of Lands cycling for monitoring and adapting the side chain composition of PC in endothelial cells.

## Data availability

An Excel spreadsheet was submitted as a Supplemental item. Other data are available from the authors on reasonable request.

## Supplemental data

This article contains supplemental data.

## Conflict of interest

The authors declare that they have no conflicts of interest with the contents of this article.

## Supplementary data

Supporting information

Supporting FigS1toS7

Supporting TablesS1toS12
