# Supplementary material for: Phosphatidylcholine synthesis and remodeling in brain endothelial cells
Source: J Lipid Res. 2025 Mar 10;66(4):100773. doi: 10.1016/j.jlr.2025.100773 (PMC12002869; doi:10.1016/j.jlr.2025.100773)
Supplement: Supporting FigS1toS7 [file mmc2.pdf]

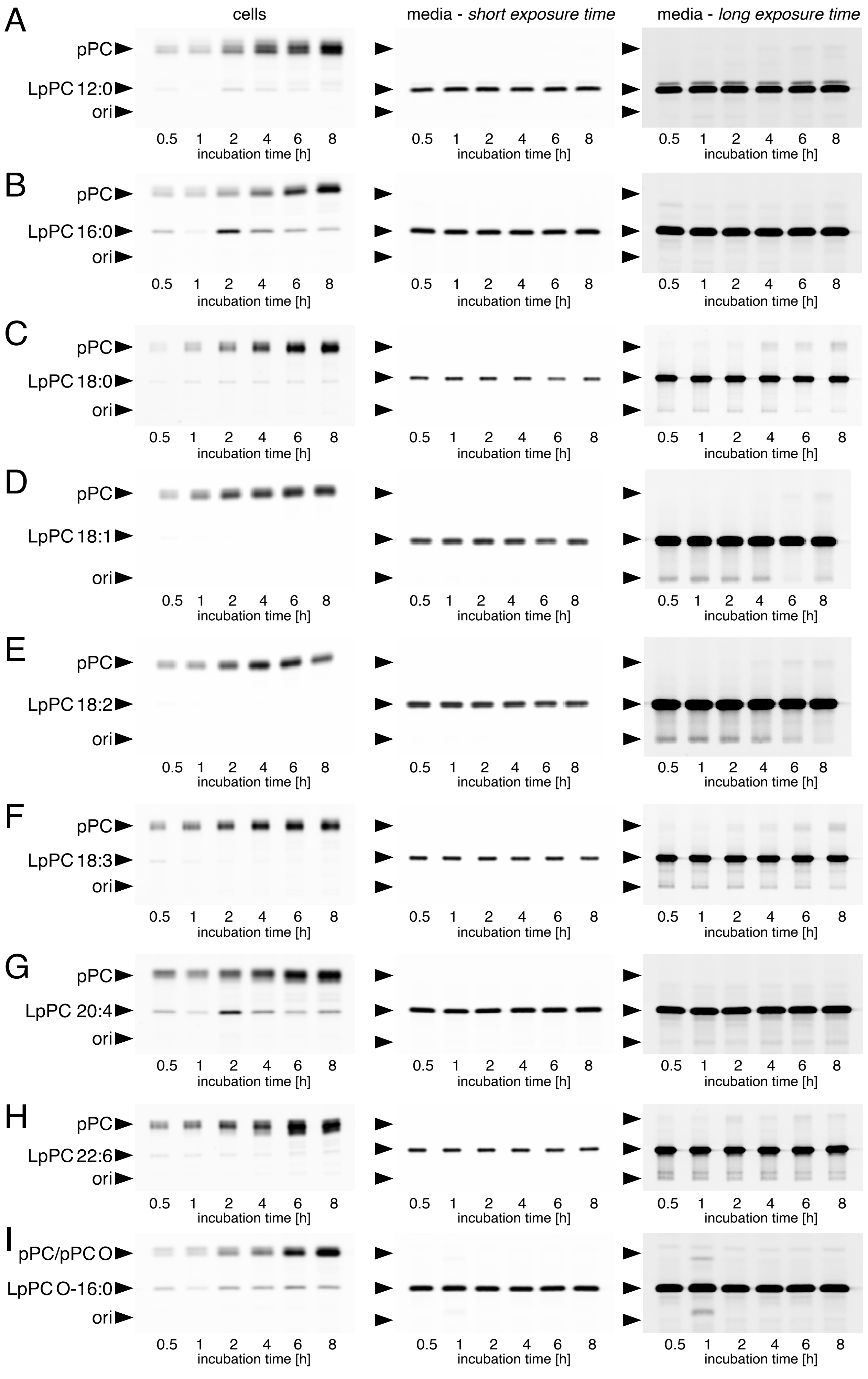

Supplemental Figure S1. TLC analysis of the kinetics of LpPC metabolism. bEND3 cells were incubated with 50 µM (A) LpPC 12:0, (B) LpPC 16:0, (C) LpPC 18:0, (D) LpPC 18:1, (E) LpPC 18:2, (F) LpPC 18:3, (G) LpPC 20:4, (H) LpPC 22:6, or (I) LpPC O-16:0 for the indicated times. Total lipids from cells (left subpanel) and incubation media (other subpanels) were isolated, click-reacted and analyzed by TLC. Images of the TLC plates analyzing the media are shown for different exposure times. Fluorescent bands were identified by co-migrating standards. Cellular pPC metabolites were detected as multiple bands. ori, origin of application.

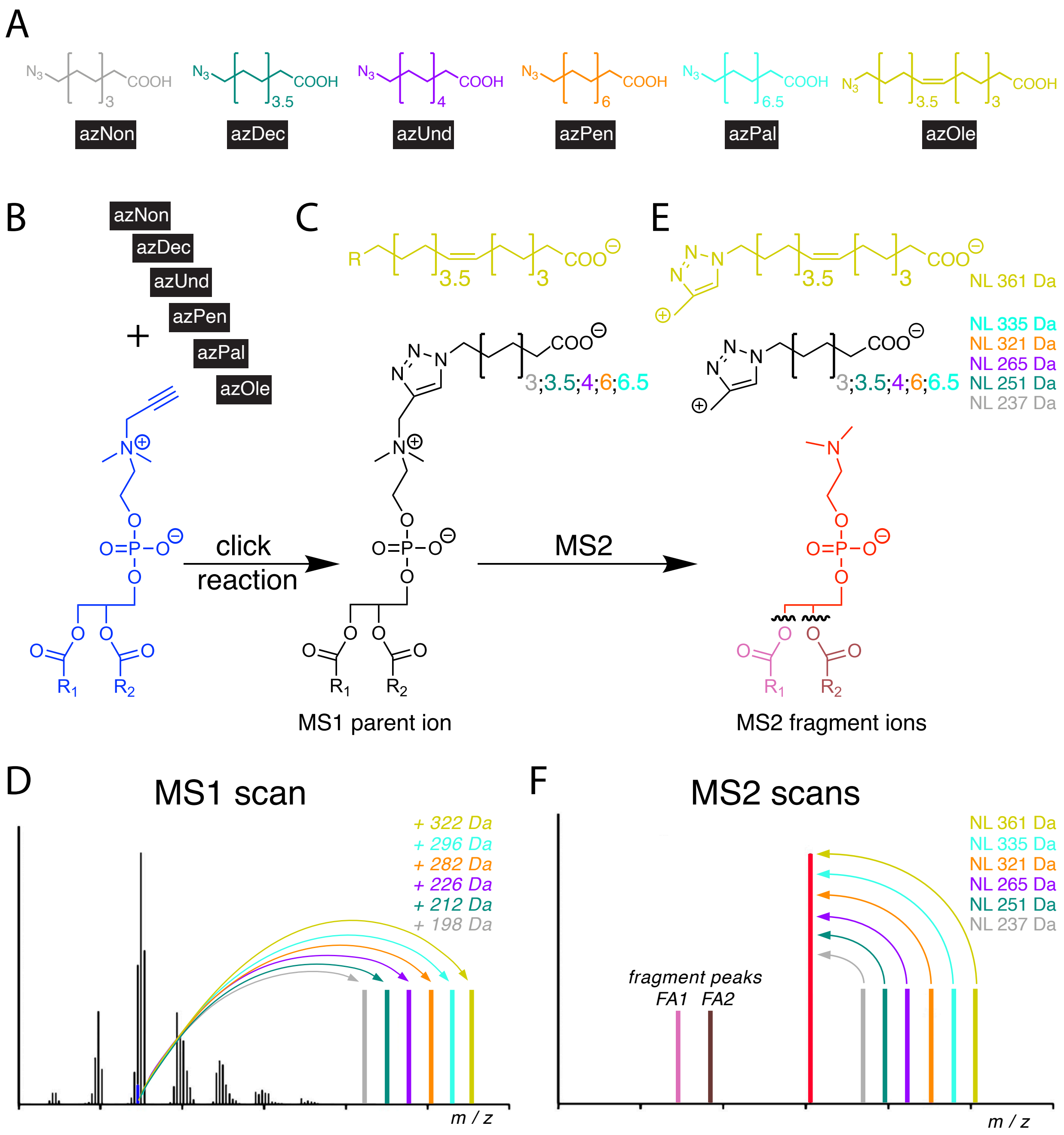

Supplemental Figure S2. Scheme of alkyne lipid detection by negative mode DIMS using azido-fatty acid reporters. (A) Structures of azido-nonanoate (azNon), azido-decanoate (azDec), azido-undecanoate (azUnd), azido-pentadecanoate (azPen), azido-palmitate (azPal) and azido-oleate (azOle). (B) For sample multiplexing one of these azido-reporters is click reacted with propargyl-labeled lipids (blue) from an individual sample before sample pooling. (C) For each analyte a mass-shifted product is generated. (D) Its MS1 ion peak is discriminated from unlabeled species of the same lipid class and from the corresponding labeled analyte of all other multiplexed samples by its uniquely mass-shifted product in MS1. (E) MS2 fragmentation yields stereotypical neutral losses (NL) that generate the same diagnostic fragment ion (F, red) that upon further fragmentation not only enables for the identification of the analyte, but also of its two FA chains.

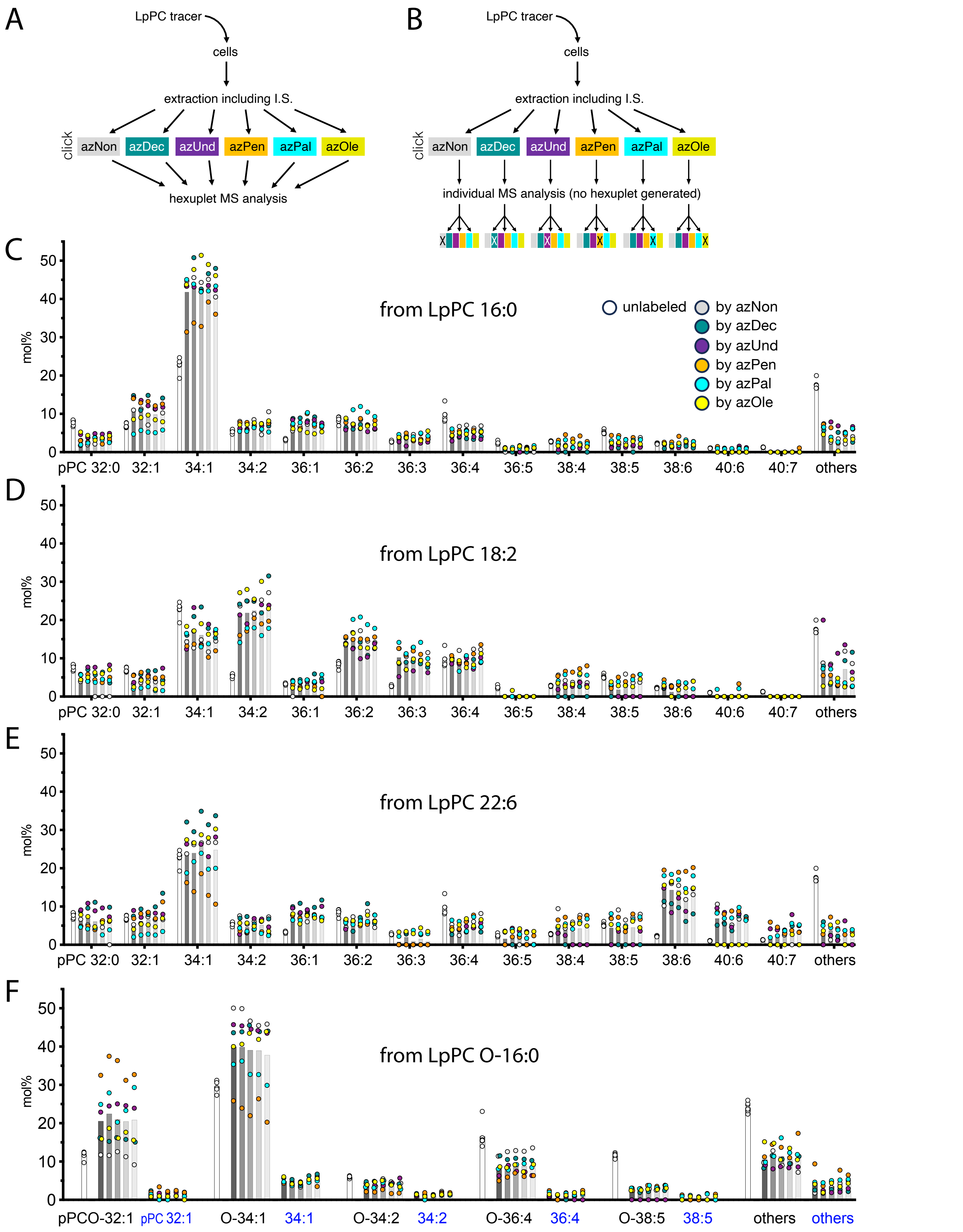

Supplemental Figure S3. Establishing hexuplet analysis of pPC metabolites. (A) bEND3 cells were incubated 50 µM LpPC tracer for 24 h. Total cellular lipids and internal standards were co-isolated and subdivided into six equal aliquots, one of which was click-reacted with either azido-nonanoate (azNon; grey symbols), azido-decanoate (azDec; teal), azido-undecanoate (azUnd; purple), azido-pentadecanoate (azPen; orange), azido-palmitate (azPal; cyan) or azido-oleate (azOle; yellow). After sample pooling MS analysis was performed on the hexuplets. (B) Pilot experiments using one azido reporter individually without hexuplet formation confirmed reliable detection of each subsample only in the expected mass range window (x), clean of crosstalk. Sample multiplexing was performed on cells incubated with (C) LpPC 16:0, (D) LpPC 18:2, (E) LpPC 22:6, or (F) LpPC O-16:0 Each biological replicate (N=5) forms one hexuplet and delivers six color-coded data points, whose average is represented as bar (grey bars). Labeled lipid species are presented as the molar fraction (mol%) of all labeled PC and PCO species. Carrier treated control samples were analyzed for quantification of the unlabeled PC and PCO pool (white symbols and bars). In (F), the transfer of the propargyl-head group from the labeled ether to the non-ether PC pool (blue) is also shown.

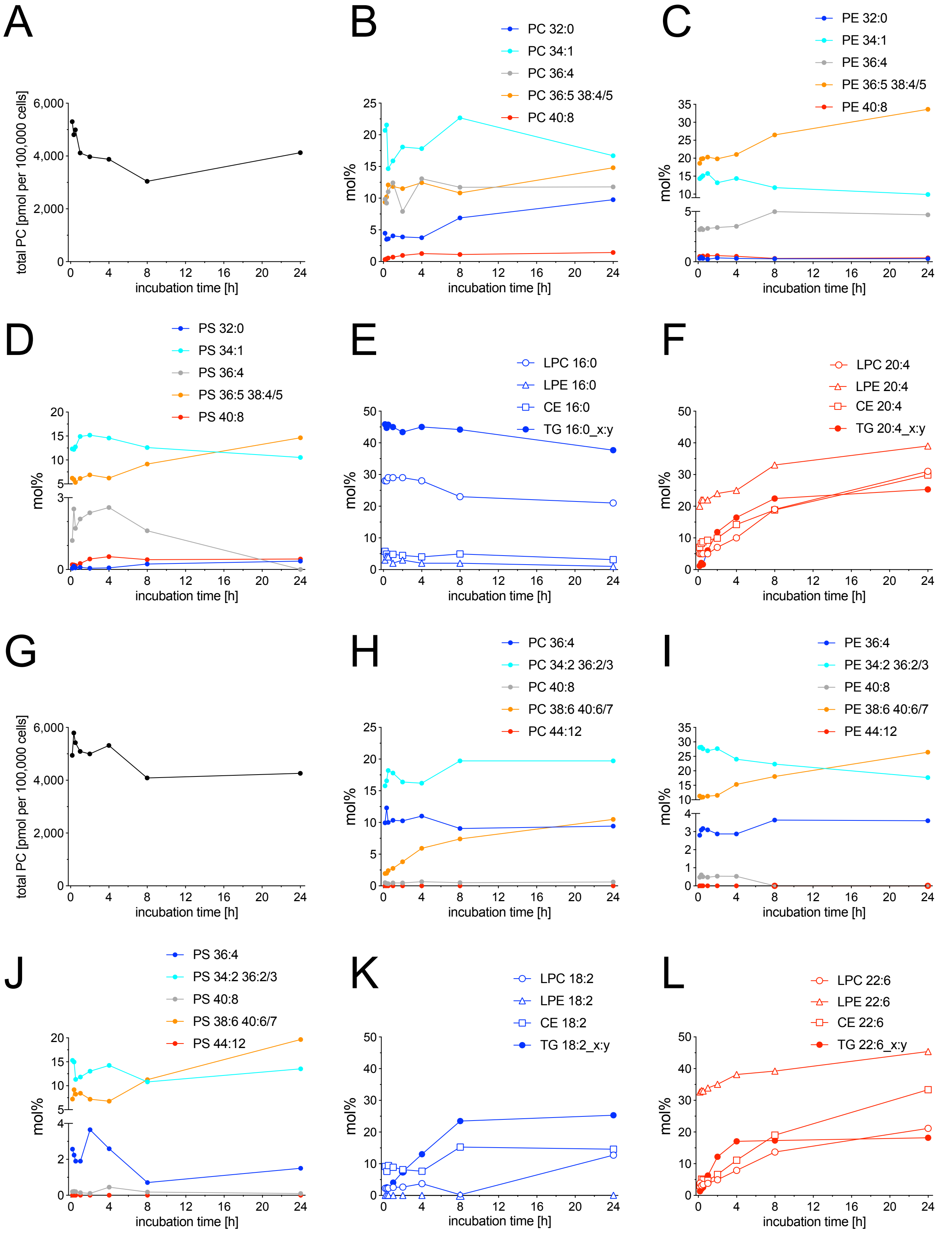

Supplemental Figure S4. MS analysis of unlabeled lipids in bEND3 cells. As for Fig. 2 cells were incubated with one of four LpPC tracers for various times. Total cellular lipids and internal standards were co-isolated and analyzed by MS as multiplexed samples. Unlabeled lipids of the pooled samples (A-J, multiplex of LpPC 16:0 + 20:4; G-L, multiplex of LpPC 18:2 + 22:6) were quantified over time. The concentration of total PC (A,G) and the molar fraction (mol%) of selected species of the respective lipid class (B-F, H-L) are presented. The analysis of PC (A,B,G,H), PE (C,I), PS (D,J) did not provide molecular species resolution. Lipid species likely to contain the poly-unsaturated FA 20:4 or FA 22:6 are depicted in red or orange; those likely containing FA 16:0 or FA 18:2 are shown in blue or cyan. For LPC, LPE, CE and TG (E,F,K,L) one acyl chain was identified.

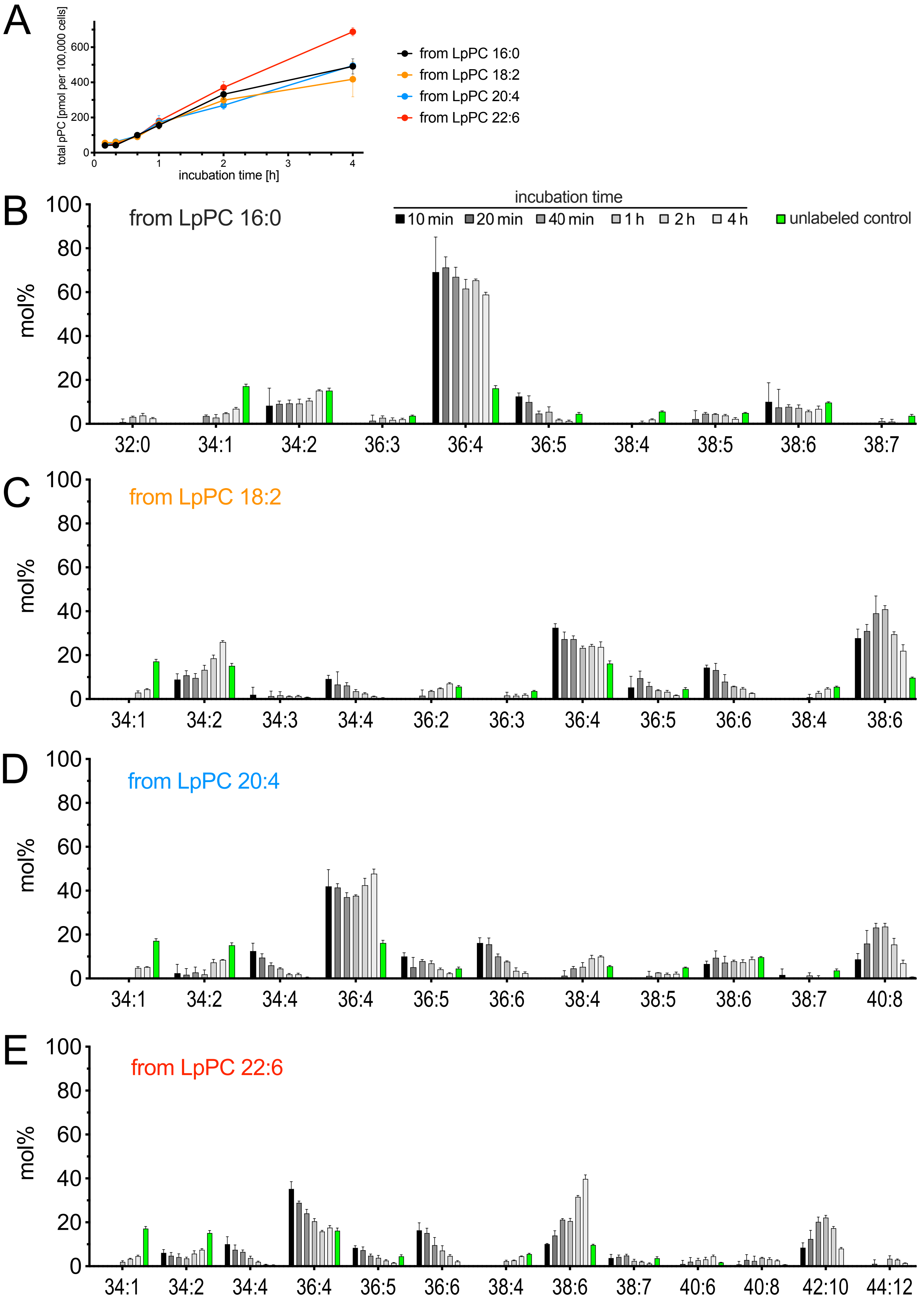

Supplemental Figure S5. MS analysis of hepatocyte pPC metabolism over time. Primary hepatocytes were incubated with one of four LpPC tracers (50 μM) for various times. Total cellular lipids and internal standards were co-isolated and click-reacted before MS analysis. All labeled pPC species were quantified (see Supplemental Tables S5-S8). (A) The total cellular pPC content per 100,000 cells is plotted over time. Error bars smaller than symbol size are omitted; N=3. Cells incubated with (B) LpPC 16:0, (C) LpPC 18:2, (D) LpPC 20:4, or (E) LpPC 22:6 showed distinct distributions of labeled pPC species. Main labeled pPC species are presented as the molar fraction (mol%) of all labeled pPC. The species distribution of unlabeled control samples (green bars, N=5) is also shown.

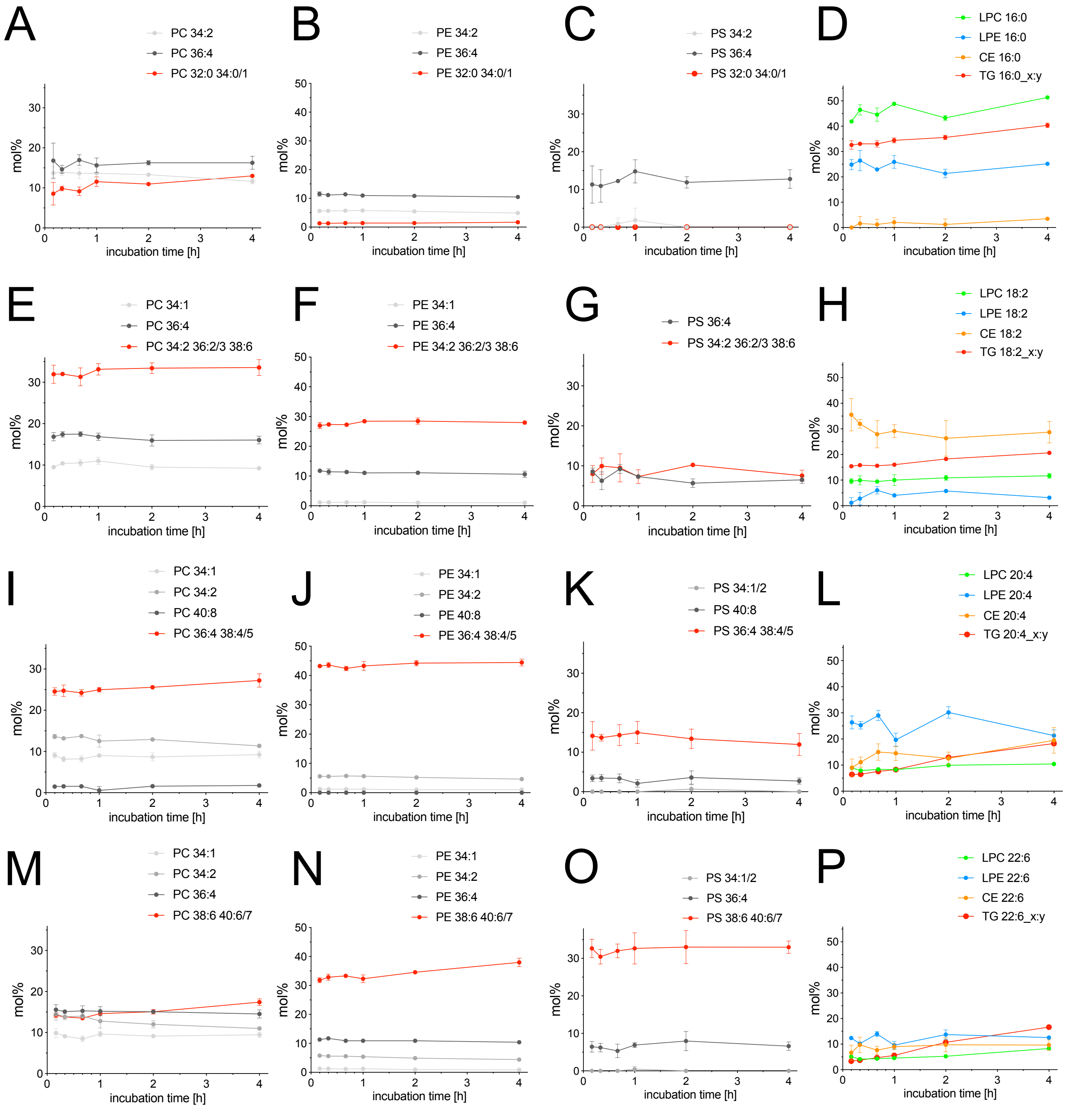

Supplemental Figure S6. MS analysis of unlabeled lipids in hepatocytes. As for Supplemental Fig. S5 cells were incubated with one of four LpPC tracers for various times. Total cellular lipids and internal standards were co-isolated and analyzed by MS. Unlabeled lipids after incubation with (A-D) LpPC 16:0, (E-H) LpPC 18:2, (I-L) LpPC 20:4, or (M-P) LpPC 22:6 were quantified over time and selected species are presented as the molar fraction (mol%) of the respective lipid class. The analysis of PC (A,E,I,M), PE (B,F,J,N) and PS (C,G,K,O) did not provide molecular species resolution and species likely to contain the acyl chain of the input tracer are depicted in red. For LPC, LPE, CE and TG (D,H,L,P) one acyl chain was identified. Data are mean; N=3. Error bars smaller than symbol size are omitted.

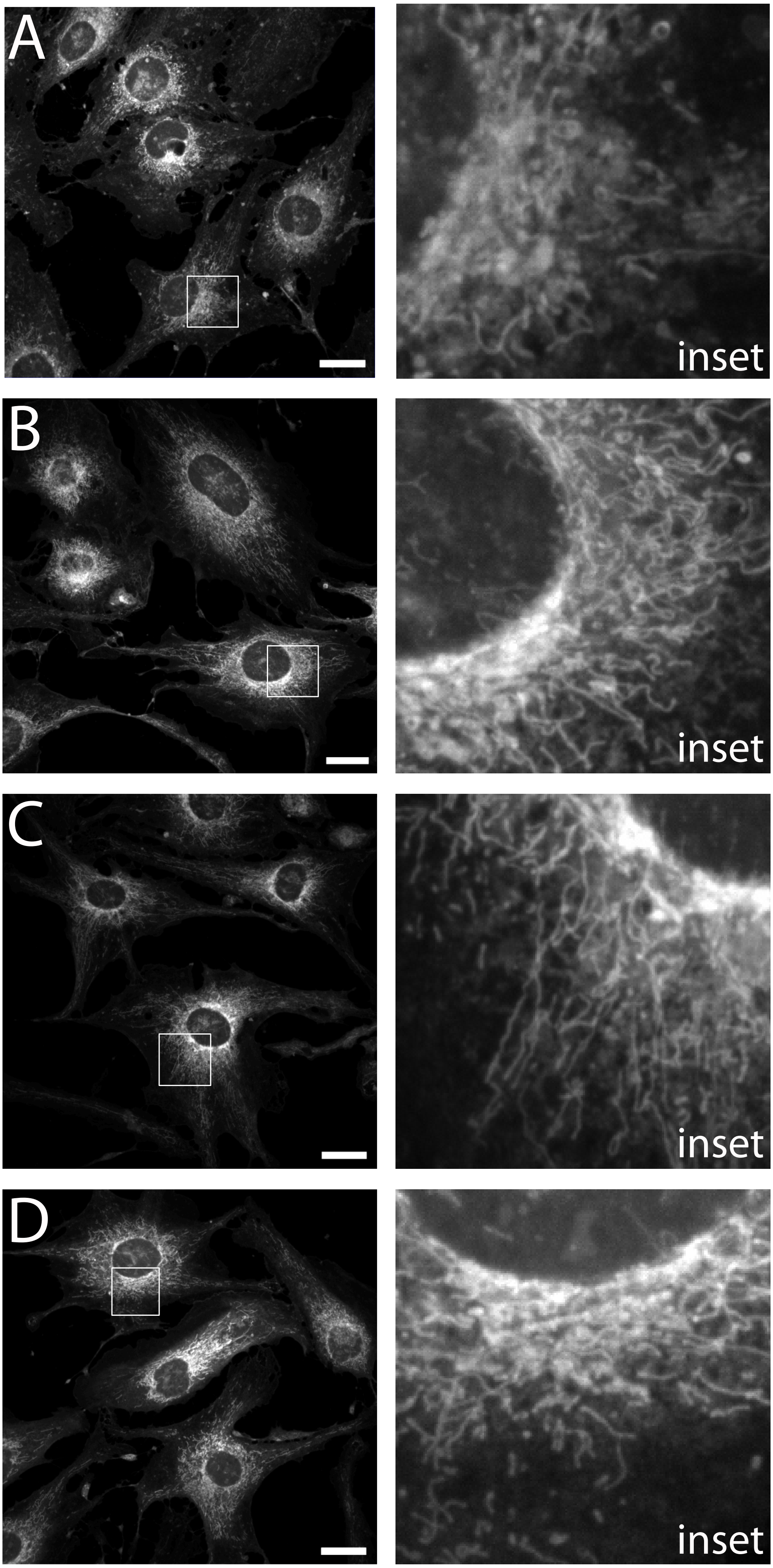

Supplemental Figure S7. Cellular distribution of pPC in bEND3 cells. Cells were incubated with 50 µM of (A) LpPC 16:0, (B) LpPC 18:2, (C) LpPC 20:4, or (D) LpPC 22:6 for 10 min. After fixation the alkyne lipids were click-reacted with fluorescent Alexa488-reporter azide. Micrographs were recorded using structured illumination microscopy. Maximum image projections of z-stacks are depicted. Bars, 20 µm.
