# Supplementary material for: Phosphatidylcholine synthesis and remodeling in brain endothelial cells
Source: J Lipid Res. 2025 Mar 10;66(4):100773. doi: 10.1016/j.jlr.2025.100773 (PMC12002869; doi:10.1016/j.jlr.2025.100773)
Supplement: Supporting TablesS1toS12 [file mmc3.pdf]

SUPPLEMENTAL TABLE S1. Species distribution of the propargylcholine-labeled PC in bEND3 cells upon incubation with LpPC 16:0. Total lipids isolated from cells labeled with 50 µM of tracer for the indicated time were analyzed by MS as multiplexed samples. Labeled lipid species were identified by their specific NL peak and quantified using synthetic pPC 31:1 as internal standard. Molecular species were identified by the FA peaks but omitted from table for clarity. Only the fractions of selected molecular species are shown (*italics*); no *sn*-position information available. Species amounts are shown as pmol per 100,000 cells and represent means ± SD; N=3.

	incubation time							
pPC	10 min	20 min	30 min	60 min	2 h	4 h	8 h	24 h
30:0	0.7 ± 0.1	1.3 ± 0.3	2.2 ± 0.6	3.2 ± 0.4	3.2 ± 0.5	3.9 ± 0.2	3.4 ± 0.7	2.5 ± 0.2
30:1	0.5 ± 0.2	0.6 ± 0.2	0.8 ± 0.2	0.7 ± 0.2	0.6 ± 0.2	0.7 ± 0.1	0.7 ± 0.3	0.6 ± 0.2
32:0	6.2 ± 0.9	12.0 ± 1.5	18.9 ± 3.3	28.2 ± 4.1	26.4 ± 2.5	28.8 ± 2.6	25.9 ± 4.7	19.6 ± 1.2
14:0_18:0	*2 %*	*2 %*	*2 %*	*0 %*	*4 %*	*1 %*	*11 %*	*9 %*
16:0_16:0	*98 %*	*98 %*	*98 %*	*100 %*	*96 %*	*99 %*	*89 %*	*91 %*
32:1	4.5 ± 1.4	6.2 ± 1.1	10.1 ± 1.6	17.7 ± 2.8	24.0 ± 3.6	41.5 ± 6.4	73.7 ± 4.9	51.4 ± 5.7
32:2	1.2 ± 0.6	1.1 ± 0.5	1.4 ± 0.6	1.5 ± 0.7	1.3 ± 0.4	1.6 ± 0.4	1.8 ± 0.4	1.6 ± 0.1
32:3	0.0 ± 0.0	0.0 ± 0.0	0.0 ± 0.0	0.0 ± 0.0	0.0 ± 0.0	0.0 ± 0.0	0.0 ± 0.0	0.0 ± 0.0
34:0	1.1 ± 0.1	1.5 ± 0.6	1.9 ± 0.3	2.4 ± 0.4	2.1 ± 0.7	2.2 ± 0.5	1.2 ± 0.4	0.8 ± 0.2
34:1	8.3 ± 0.3	13.2 ± 0.4	19.5 ± 1.9	41.1 ± 3.8	64.6 ± 2.4	114.1 ± 9.1	196.3 ± 12.7	202.1 ± 35.3
34:2	4.5 ± 1.6	5.0 ± 1.4	6.4 ± 1.4	8.9 ± 0.9	11.4 ± 2.0	18.0 ± 2.0	29.0 ± 4.5	28.6 ± 3.0
34:3	0.6 ± 0.5	0.8 ± 0.3	1.0 ± 0.3	1.0 ± 0.1	0.8 ± 0.2	1.5 ± 0.6	1.6 ± 0.5	1.4 ± 0.4
34:4	0.7 ± 0.2	0.4 ± 0.4	0.8 ± 0.2	0.7 ± 0.6	0.7 ± 0.3	1.0 ± 0.3	0.7 ± 0.1	0.4 ± 0.4
36:0	0.0 ± 0.0	0.0 ± 0.0	0.0 ± 0.0	0.0 ± 0.0	0.6 ± 0.1	0.7 ± 0.2	0.2 ± 0.2	0.0 ± 0.0
36:1	1.0 ± 0.3	1.2 ± 0.4	2.3 ± 0.1	3.9 ± 0.6	5.8 ± 0.8	9.7 ± 1.3	13.1 ± 2.6	21.5 ± 4.4
36:2	1.1 ± 0.4	1.7 ± 0.5	2.4 ± 0.2	3.8 ± 0.6	6.2 ± 1.5	11.3 ± 1.9	18.5 ± 2.1	30.6 ± 8.2
36:3	1.8 ± 0.4	1.8 ± 0.1	3.2 ± 0.3	4.7 ± 0.5	7.8 ± 0.5	12.6 ± 1.0	17.9 ± 3.5	13.6 ± 2.6
36:4	7.2 ± 1.1	9.8 ± 2.1	14.5 ± 1.7	22.3 ± 2.8	31.2 ± 1.3	43.7 ± 4.4	53.0 ± 5.3	23.4 ± 7.5
36:5	1.6 ± 0.6	2.2 ± 0.1	3.3 ± 0.3	4.0 ± 0.3	5.1 ± 0.3	7.2 ± 0.7	7.1 ± 0.7	4.3 ± 1.0
36:6	0.3 ± 0.3	0.4 ± 0.3	0.5 ± 0.1	0.5 ± 0.5	0.4 ± 0.4	0.6 ± 0.6	0.4 ± 0.3	0.2 ± 0.3
38:0	0.0 ± 0.0	0.1 ± 0.3	0.2 ± 0.3	0.7 ± 0.2	0.9 ± 0.4	1.2 ± 0.1	0.8 ± 0.4	0.2 ± 0.4
38:1	0.4 ± 0.4	0.8 ± 0.4	1.0 ± 0.3	1.7 ± 0.3	2.3 ± 0.7	2.7 ± 0.4	1.9 ± 0.3	2.9 ± 1.1
38:2	0.0 ± 0.0	0.2 ± 0.4	0.0 ± 0.0	0.2 ± 0.4	0.6 ± 0.5	1.5 ± 1.3	1.2 ± 1.1	3.6 ± 2.8
38:3	0.4 ± 0.3	0.4 ± 0.3	0.8 ± 0.1	0.8 ± 0.1	1.0 ± 0.2	2.0 ± 0.3	2.2 ± 0.3	3.9 ± 0.9
38:4	1.2 ± 0.3	1.1 ± 0.2	2.2 ± 0.5	2.5 ± 0.5	3.3 ± 0.5	5.8 ± 0.6	7.1 ± 1.4	9.7 ± 2.2
38:5	2.8 ± 0.7	3.3 ± 0.7	6.2 ± 1.4	7.7 ± 0.1	8.8 ± 1.7	13.1 ± 1.7	15.8 ± 4.4	10.6 ± 2.6
38:6	3.9 ± 0.2	5.6 ± 0.7	7.3 ± 0.5	10.7 ± 1.5	11.8 ± 0.5	17.7 ± 2.4	22.5 ± 4.4	10.1 ± 3.4
38:7	0.4 ± 0.3	0.2 ± 0.3	0.5 ± 0.5	0.3 ± 0.4	0.0 ± 0.0	0.2 ± 0.3	0.4 ± 0.3	0.6 ± 0.1
40:0	0.0 ± 0.0	0.0 ± 0.0	0.3 ± 0.3	0.8 ± 0.3	1.3 ± 0.4	1.3 ± 1.0	0.4 ± 0.3	0.0 ± 0.0
40:1	0.2 ± 0.3	0.9 ± 0.2	0.2 ± 0.4	1.3 ± 0.0	1.5 ± 0.3	2.1 ± 0.3	2.1 ± 0.6	5.0 ± 2.6
40:2	0.0 ± 0.0	0.0 ± 0.0	0.2 ± 0.3	0.4 ± 0.4	0.7 ± 0.2	1.8 ± 0.6	1.5 ± 0.4	4.0 ± 1.9
40:3	0.0 ± 0.0	0.0 ± 0.0	0.0 ± 0.0	0.0 ± 0.0	0.0 ± 0.0	0.4 ± 0.4	0.3 ± 0.3	0.9 ± 0.2
40:4	0.0 ± 0.0	0.2 ± 0.3	0.0 ± 0.0	0.1 ± 0.2	0.2 ± 0.4	0.9 ± 0.1	0.9 ± 0.2	1.4 ± 0.4
40:5	0.1 ± 0.2	0.0 ± 0.0	0.4 ± 0.3	0.9 ± 0.3	0.8 ± 0.3	1.3 ± 0.1	1.3 ± 0.1	2.7 ± 0.8
40:6	0.0 ± 0.0	0.0 ± 0.0	0.6 ± 0.6	0.8 ± 0.1	1.3 ± 0.4	1.1 ± 0.1	1.8 ± 0.3	3.1 ± 0.8
40:7	0.2 ± 0.4	0.3 ± 0.3	0.9 ± 0.1	0.8 ± 0.1	1.1 ± 0.3	1.9 ± 0.1	2.2 ± 0.7	2.1 ± 0.5
40:8	0.0 ± 0.0	0.0 ± 0.0	0.0 ± 0.0	0.2 ± 0.4	0.0 ± 0.0	0.0 ± 0.0	0.0 ± 0.0	0.0 ± 0.0
42:1	0.0 ± 0.0	0.0 ± 0.0	0.0 ± 0.0	0.0 ± 0.0	0.4 ± 0.4	0.8 ± 0.7	1.0 ± 0.9	2.2 ± 2.0
42:2	0.0 ± 0.0	0.0 ± 0.0	0.0 ± 0.0	0.0 ± 0.0	0.7 ± 0.2	1.4 ± 0.2	1.2 ± 0.3	3.0 ± 1.0
42:3	0.0 ± 0.0	0.0 ± 0.0	0.0 ± 0.0	0.0 ± 0.0	0.0 ± 0.0	0.0 ± 0.0	0.0 ± 0.0	0.3 ± 0.5
42:4	0.0 ± 0.0	0.0 ± 0.0	0.0 ± 0.0	0.2 ± 0.3	0.0 ± 0.0	0.6 ± 0.6	0.9 ± 0.1	0.7 ± 0.1
42:5	0.0 ± 0.0	0.0 ± 0.0	0.0 ± 0.0	0.0 ± 0.0	0.0 ± 0.0	0.5 ± 0.4	0.0 ± 0.0	0.3 ± 0.5
44:4	0.0 ± 0.0	0.0 ± 0.0	0.0 ± 0.0	0.0 ± 0.0	0.1 ± 0.2	0.7 ± 0.1	0.3 ± 0.3	0.7 ± 0.4
44:5	0.0 ± 0.0	0.0 ± 0.0	0.0 ± 0.0	0.0 ± 0.0	0.0 ± 0.0	0.8 ± 0.7	0.4 ± 0.4	0.6 ± 0.1
46:6	0.0 ± 0.0	0.0 ± 0.0	0.0 ± 0.0	0.0 ± 0.0	0.0 ± 0.0	0.0 ± 0.0	0.0 ± 0.0	0.4 ± 0.3
**total**	**51.0 ± 9.2**	**72.5 ± 4.7**	**110.2 ± 5.4**	**174.7 ± 11.1**	**229.3 ± 5.5**	**359.2 ± 24.5**	**510.9 ± 41.7**	**471.6 ± 80.8**

SUPPLEMENTAL TABLE S2. Species distribution of the propargylcholine-labeled PC in bEND3 cells upon incubation with LpPC 18:2. Total lipids isolated from cells labeled with 50 µM of tracer for the indicated time were analyzed by MS as multiplexed samples. Labeled lipid species were identified by their specific NL peak and quantified using synthetic pPC 31:1 as internal standard. Molecular species were identified by the FA peaks but omitted from table for clarity. Only the fractions of selected molecular species are shown (*italics*); no *sn*-position information available. Species amounts are shown as pmol per 100,000 cells and represent means ± SD; N=3.

	incubation time							
pPC	10 min	20 min	30 min	60 min	2 h	4 h	8 h	24 h
30:0	0.0 ± 0.0	0.0 ± 0.0	0.0 ± 0.0	0.0 ± 0.0	0.0 ± 0.0	0.2 ± 0.3	1.1 ± 0.2	1.2 ± 0.1
30:1	0.3 ± 0.3	0.3 ± 0.5	0.3 ± 0.4	0.3 ± 0.3	0.3 ± 0.4	0.6 ± 0.2	0.3 ± 0.3	0.1 ± 0.2
32:0	0.0 ± 0.0	0.0 ± 0.0	0.2 ± 0.3	0.2 ± 0.4	0.8 ± 0.3	1.6 ± 0.3	3.8 ± 0.8	11.3 ± 0.9
32:1	1.8 ± 0.9	2.1 ± 1.5	1.7 ± 0.8	2.6 ± 1.7	3.1 ± 1.2	4.4 ± 1.1	5.9 ± 0.4	8.5 ± 0.9
32:2	1.1 ± 0.1	1.2 ± 0.4	1.5 ± 0.7	1.8 ± 0.8	1.7 ± 0.6	2.1 ± 0.5	1.5 ± 0.4	1.2 ± 0.2
32:3	0.0 ± 0.0	0.0 ± 0.0	0.0 ± 0.0	0.0 ± 0.0	0.0 ± 0.0	0.0 ± 0.0	0.0 ± 0.0	0.0 ± 0.0
34:0	0.3 ± 0.5	0.2 ± 0.4	0.3 ± 0.5	0.4 ± 0.7	0.0 ± 0.0	0.4 ± 0.7	0.7 ± 0.6	2.0 ± 0.5
34:1	1.4 ± 1.1	1.7 ± 0.9	1.7 ± 1.2	2.4 ± 1.5	4.7 ± 1.7	9.3 ± 2.2	15.4 ± 4.3	31.8 ± 4.7
34:2	4.1 ± 1.9	6.2 ± 2.3	9.5 ± 2.8	14.3 ± 2.2	15.8 ± 3.7	19.6 ± 2.4	28.8 ± 6.4	41.3 ± 2.8
34:3	1.7 ± 0.2	2.8 ± 0.3	4.6 ± 0.9	7.5 ± 0.9	9.5 ± 1.8	8.9 ± 0.7	6.9 ± 1.3	4.2 ± 0.6
34:4	0.5 ± 0.5	0.3 ± 0.5	0.3 ± 0.5	0.3 ± 0.5	0.2 ± 0.4	0.6 ± 0.5	0.8 ± 0.1	0.2 ± 0.3
36:0	0.0 ± 0.0	0.0 ± 0.0	0.0 ± 0.0	0.0 ± 0.0	0.0 ± 0.0	0.0 ± 0.0	0.1 ± 0.2	0.4 ± 0.3
36:1	0.3 ± 0.3	0.3 ± 0.3	0.1 ± 0.1	0.2 ± 0.3	0.6 ± 0.6	1.9 ± 0.5	2.5 ± 0.1	6.4 ± 1.3
36:2	2.2 ± 1.1	4.1 ± 1.0	6.8 ± 2.0	10.4 ± 1.4	14.4 ± 2.0	21.2 ± 1.9	33.1 ± 4.3	31.4 ± 2.7
36:3	4.0 ± 2.0	6.3 ± 1.4	12.9 ± 2.6	17.2 ± 3.2	25.0 ± 2.0	31.6 ± 2.8	29.8 ± 4.2	18.2 ± 1.8
36:4	9.1 ± 2.2	15.1 ± 4.8	16.8 ± 4.3	33.5 ± 6.7	47.2 ± 10.3	62.5 ± 8.8	63.5 ± 12.0	20.6 ± 7.1
14:0_22:4	*9 %*	*8 %*	*5 %*	*4 %*	*3 %*	*2 %*	*2 %*	*4 %*
16:0_20:4	*18 %*	*14 %*	*13 %*	*10 %*	*8 %*	*9 %*	*11 %*	*34 %*
16:1_20:3	*17 %*	*15 %*	*12 %*	*9 %*	*7 %*	*5 %*	*4 %*	*7 %*
18:2_18:2	*41 %*	*52 %*	*60 %*	*69 %*	*78 %*	*80 %*	*81 %*	*50 %*
18:3_18:1	*15 %*	*11 %*	*10 %*	*7 %*	*5 %*	*4 %*	*2 %*	*4 %*
14:3_22:1	*1 %*	*1 %*	*1 %*	*0 %*	*0 %*	*0 %*	*0 %*	*0 %*
36:5	0.8 ± 0.9	1.1 ± 0.8	0.8 ± 0.9	1.5 ± 0.8	1.0 ± 0.2	1.8 ± 0.6	1.4 ± 0.6	1.6 ± 0.5
36:6	0.0 ± 0.0	0.2 ± 0.4	0.0 ± 0.0	0.3 ± 0.6	0.5 ± 0.4	0.7 ± 0.6	0.2 ± 0.3	0.4 ± 0.7
38:0	0.0 ± 0.0	0.0 ± 0.0	0.0 ± 0.0	0.0 ± 0.0	0.0 ± 0.0	0.0 ± 0.0	0.0 ± 0.0	0.8 ± 0.1
38:1	0.0 ± 0.0	0.1 ± 0.2	0.0 ± 0.0	0.1 ± 0.2	0.1 ± 0.2	0.2 ± 0.4	0.8 ± 0.2	1.4 ± 0.2
38:2	0.3 ± 0.3	0.1 ± 0.1	0.4 ± 0.3	0.4 ± 0.4	1.1 ± 0.2	2.9 ± 0.7	1.5 ± 0.3	2.8 ± 0.4
38:3	0.7 ± 0.0	0.8 ± 0.2	1.6 ± 0.2	1.7 ± 0.6	2.6 ± 0.3	5.3 ± 0.4	3.7 ± 0.5	4.2 ± 0.7
38:4	1.1 ± 0.7	0.8 ± 0.1	0.6 ± 0.7	1.7 ± 0.5	2.2 ± 0.5	5.2 ± 0.3	7.9 ± 1.3	10.6 ± 1.0
38:5	1.8 ± 0.1	2.3 ± 0.0	3.2 ± 0.5	4.3 ± 0.6	6.2 ± 0.7	7.4 ± 0.4	7.7 ± 1.2	6.7 ± 1.1
38:6	6.2 ± 1.0	10.7 ± 1.0	13.8 ± 0.4	24.2 ± 2.0	29.6 ± 2.5	40.5 ± 4.1	35.7 ± 3.3	9.1 ± 1.3
38:7	0.9 ± 0.1	1.5 ± 0.6	2.5 ± 0.5	3.8 ± 0.8	4.6 ± 0.6	4.1 ± 0.6	3.5 ± 0.4	0.6 ± 0.7
40:0	0.0 ± 0.0	0.0 ± 0.0	0.0 ± 0.0	0.0 ± 0.0	0.0 ± 0.0	0.0 ± 0.0	0.0 ± 0.0	0.3 ± 0.5
40:1	0.0 ± 0.0	0.0 ± 0.0	0.0 ± 0.0	0.0 ± 0.0	0.2 ± 0.3	0.7 ± 0.6	0.4 ± 0.3	2.1 ± 0.4
40:2	0.4 ± 0.4	0.5 ± 0.5	0.8 ± 0.7	1.5 ± 0.1	2.1 ± 0.3	5.2 ± 1.6	3.5 ± 0.1	4.8 ± 0.7
40:3	0.0 ± 0.0	0.5 ± 0.4	0.9 ± 0.0	2.0 ± 0.4	3.6 ± 1.2	7.0 ± 2.3	3.1 ± 0.9	4.1 ± 1.0
40:4	0.0 ± 0.0	0.0 ± 0.0	0.0 ± 0.0	0.0 ± 0.0	0.0 ± 0.0	1.7 ± 0.4	1.9 ± 0.6	2.5 ± 0.4
40:5	0.0 ± 0.0	0.0 ± 0.0	0.0 ± 0.0	0.2 ± 0.4	0.9 ± 0.1	1.5 ± 0.5	2.1 ± 0.6	3.0 ± 0.5
40:6	0.2 ± 0.3	0.4 ± 0.3	0.9 ± 0.2	1.5 ± 0.1	2.3 ± 0.6	2.6 ± 0.5	3.0 ± 0.8	3.7 ± 0.3
40:7	0.6 ± 0.5	1.4 ± 0.9	2.1 ± 0.4	4.0 ± 0.3	6.0 ± 1.5	5.2 ± 0.8	4.1 ± 1.3	2.4 ± 0.6
40:8	2.9 ± 0.2	4.6 ± 0.4	6.6 ± 0.3	8.9 ± 0.9	8.7 ± 1.1	10.5 ± 1.4	9.1 ± 1.5	1.3 ± 0.1
42:1	0.0 ± 0.0	0.0 ± 0.0	0.0 ± 0.0	0.0 ± 0.0	0.0 ± 0.0	0.0 ± 0.0	0.2 ± 0.3	1.1 ± 0.8
42:2	0.0 ± 0.0	0.6 ± 0.5	0.6 ± 0.5	1.2 ± 1.1	1.7 ± 1.5	4.0 ± 3.7	2.9 ± 2.0	3.4 ± 3.0
42:3	0.2 ± 0.3	1.1 ± 0.3	1.2 ± 0.3	1.9 ± 0.6	2.6 ± 0.3	5.9 ± 0.9	4.4 ± 0.6	4.0 ± 0.5
42:4	0.0 ± 0.0	0.0 ± 0.0	0.0 ± 0.0	0.0 ± 0.0	0.8 ± 0.8	1.6 ± 0.7	1.7 ± 0.2	3.3 ± 1.2
42:5	0.0 ± 0.0	0.0 ± 0.0	0.0 ± 0.0	0.0 ± 0.0	0.0 ± 0.0	0.5 ± 0.5	0.6 ± 0.1	1.2 ± 0.6
42:6	0.0 ± 0.0	0.0 ± 0.0	0.0 ± 0.0	0.0 ± 0.0	0.0 ± 0.0	0.0 ± 0.0	0.0 ± 0.0	1.0 ± 0.1
42:7	0.0 ± 0.0	0.0 ± 0.0	0.0 ± 0.0	0.0 ± 0.0	0.0 ± 0.0	0.0 ± 0.0	0.1 ± 0.2	0.9 ± 0.3
42:10	0.0 ± 0.0	0.3 ± 0.5	0.0 ± 0.0	0.7 ± 0.6	1.2 ± 0.6	0.0 ± 0.0	0.0 ± 0.0	0.0 ± 0.0
44:4	0.0 ± 0.0	0.0 ± 0.0	0.0 ± 0.0	0.0 ± 0.0	0.0 ± 0.0	0.3 ± 0.5	0.4 ± 0.3	1.3 ± 0.4
44:5	0.0 ± 0.0	0.0 ± 0.0	0.0 ± 0.0	0.0 ± 0.0	0.0 ± 0.0	0.0 ± 0.0	0.0 ± 0.0	1.0 ± 0.0
44:6	0.0 ± 0.0	0.0 ± 0.0	0.0 ± 0.0	0.0 ± 0.0	0.0 ± 0.0	0.0 ± 0.0	0.0 ± 0.0	0.7 ± 0.8
44:12	0.0 ± 0.0	0.5 ± 0.8	0.0 ± 0.0	0.9 ± 1.5	1.5 ± 2.5	0.0 ± 0.0	0.0 ± 0.0	0.0 ± 0.0
**total**	**42.9 ± 14.4**	**68.0 ± 16.4**	**92.5 ± 18.6**	**151.9 ± 20.3**	**202.8 ± 25.3**	**279.7 ± 23.2**	**294.2 ± 31.6**	**259.3 ± 26.6**

SUPPLEMENTAL TABLE S3. Species distribution of the propargylcholine-labeled PC in bEND3 cells upon incubation with LpPC 20:4. Total lipids isolated from cells labeled with 50 µM of tracer for the indicated time were analyzed by MS as multiplexed samples. Labeled lipid species were identified by their specific NL peak and quantified using synthetic pPC 31:1 as internal standard. Molecular species were identified by the FA peaks but omitted from table for clarity. Only the fractions of selected molecular species are shown (*italics*); no *sn*-position information available. Species amounts are shown as pmol per 100,000 cells and represent means ± SD; N=3.

	incubation time							
pPC	10 min	20 min	30 min	60 min	2 h	4 h	8 h	24 h
30:0	0.0 ± 0.0	0.0 ± 0.0	0.2 ± 0.3	0.1 ± 0.2	0.0 ± 0.0	0.4 ± 0.4	1.0 ± 0.3	2.9 ± 0.7
30:1	0.1 ± 0.2	0.0 ± 0.0	0.3 ± 0.3	0.0 ± 0.0	0.0 ± 0.0	0.3 ± 0.3	0.2 ± 0.3	0.3 ± 0.3
32:0	0.0 ± 0.0	0.3 ± 0.3	0.5 ± 0.4	1.2 ± 0.3	0.9 ± 0.3	2.8 ± 0.5	5.9 ± 1.1	19.1 ± 2.5
32:1	1.3 ± 0.8	1.6 ± 0.9	2.2 ± 1.4	2.5 ± 1.1	3.1 ± 0.8	4.9 ± 0.4	6.7 ± 0.2	13.0 ± 0.2
32:2	0.6 ± 0.3	0.5 ± 0.2	0.9 ± 0.6	0.8 ± 0.2	0.7 ± 0.7	1.1 ± 0.3	1.0 ± 0.3	1.2 ± 0.2
32:3	0.0 ± 0.0	0.0 ± 0.0	0.0 ± 0.0	0.0 ± 0.0	0.0 ± 0.0	0.0 ± 0.0	0.0 ± 0.0	0.0 ± 0.0
34:0	0.4 ± 0.6	0.2 ± 0.3	0.0 ± 0.0	0.5 ± 0.4	0.3 ± 0.5	0.9 ± 0.1	1.5 ± 0.7	2.9 ± 1.0
34:1	1.2 ± 0.7	1.6 ± 1.3	1.8 ± 0.6	3.6 ± 0.8	4.6 ± 1.2	9.4 ± 1.1	19.4 ± 2.8	51.8 ± 3.8
34:2	1.9 ± 1.3	2.1 ± 1.5	3.1 ± 2.1	2.7 ± 1.9	3.0 ± 1.3	4.8 ± 1.6	5.4 ± 1.1	7.2 ± 0.9
34:3	0.2 ± 0.4	0.4 ± 0.3	0.3 ± 0.6	0.3 ± 0.5	0.5 ± 0.5	0.2 ± 0.4	0.5 ± 0.4	0.9 ± 0.1
34:4	0.4 ± 0.4	0.5 ± 0.5	1.0 ± 0.2	0.8 ± 0.7	1.2 ± 0.3	0.9 ± 0.0	1.6 ± 1.0	1.7 ± 0.8
36:0	0.0 ± 0.0	0.0 ± 0.0	0.0 ± 0.0	0.0 ± 0.0	0.0 ± 0.0	0.0 ± 0.0	0.0 ± 0.0	0.3 ± 0.5
36:1	0.2 ± 0.2	0.3 ± 0.4	0.2 ± 0.3	0.7 ± 0.1	1.1 ± 0.4	2.5 ± 1.0	4.4 ± 0.9	8.3 ± 2.1
36:2	0.6 ± 0.6	0.5 ± 0.5	1.0 ± 0.5	1.5 ± 0.9	2.3 ± 0.7	4.4 ± 0.7	6.4 ± 0.9	9.4 ± 2.7
36:3	0.7 ± 0.5	0.5 ± 0.8	1.0 ± 0.9	0.9 ± 0.5	1.1 ± 0.5	2.3 ± 0.6	2.7 ± 1.2	3.2 ± 1.8
36:4	3.6 ± 1.3	4.8 ± 1.8	7.5 ± 2.5	10.8 ± 4.2	11.5 ± 1.9	17.3 ± 3.0	20.6 ± 4.2	37.9 ± 7.5
36:5	2.0 ± 0.5	2.5 ± 0.6	4.4 ± 1.7	6.3 ± 0.7	6.5 ± 0.7	7.1 ± 0.8	5.0 ± 0.9	4.1 ± 0.4
36:6	0.3 ± 0.4	0.2 ± 0.3	0.2 ± 0.4	0.4 ± 0.7	0.5 ± 0.4	0.2 ± 0.4	0.3 ± 0.6	0.2 ± 0.4
38:0	0.0 ± 0.0	0.0 ± 0.0	0.0 ± 0.0	0.0 ± 0.0	0.0 ± 0.0	0.0 ± 0.0	0.0 ± 0.0	1.1 ± 0.2
38:1	0.0 ± 0.0	0.1 ± 0.1	0.0 ± 0.0	0.1 ± 0.1	0.1 ± 0.2	0.5 ± 0.1	0.1 ± 0.2	1.8 ± 0.5
38:2	0.2 ± 0.3	0.1 ± 0.1	0.0 ± 0.0	0.1 ± 0.1	0.1 ± 0.2	0.7 ± 0.6	0.8 ± 0.1	1.2 ± 0.2
38:3	0.0 ± 0.0	0.0 ± 0.0	0.3 ± 0.6	0.3 ± 0.2	0.4 ± 0.4	1.0 ± 0.1	0.9 ± 0.2	1.6 ± 0.6
38:4	3.0 ± 1.1	4.6 ± 0.6	9.5 ± 0.6	12.3 ± 0.3	20.2 ± 1.2	35.6 ± 5.6	51.2 ± 3.0	51.9 ± 3.8
38:5	3.0 ± 0.6	5.2 ± 1.9	8.1 ± 6.3	16.1 ± 2.1	19.4 ± 0.9	30.1 ± 3.5	28.1 ± 0.6	22.7 ± 1.9
38:6	1.4 ± 0.4	1.5 ± 0.3	3.1 ± 0.4	3.2 ± 1.1	3.9 ± 0.7	4.3 ± 0.7	4.0 ± 0.6	3.4 ± 0.7
38:7	0.0 ± 0.0	0.0 ± 0.0	0.0 ± 0.0	0.1 ± 0.2	0.2 ± 0.4	0.6 ± 0.5	0.2 ± 0.3	0.5 ± 0.4
40:0	0.0 ± 0.0	0.0 ± 0.0	0.0 ± 0.0	0.0 ± 0.0	0.0 ± 0.0	0.0 ± 0.0	0.0 ± 0.0	1.3 ± 0.7
40:1	0.1 ± 0.3	0.0 ± 0.0	0.0 ± 0.0	0.0 ± 0.0	0.0 ± 0.0	0.8 ± 0.2	0.7 ± 0.1	1.6 ± 0.6
40:2	0.0 ± 0.0	0.0 ± 0.0	0.0 ± 0.0	0.1 ± 0.2	0.1 ± 0.2	0.9 ± 0.4	1.1 ± 0.1	1.1 ± 0.5
40:3	0.0 ± 0.0	0.0 ± 0.0	0.0 ± 0.0	0.0 ± 0.0	0.0 ± 0.0	0.0 ± 0.0	0.0 ± 0.0	0.0 ± 0.0
40:4	0.3 ± 0.3	0.2 ± 0.3	0.4 ± 0.7	0.2 ± 0.4	0.7 ± 0.2	1.9 ± 0.5	3.6 ± 1.1	10.6 ± 1.1
40:5	0.2 ± 0.4	0.4 ± 0.3	0.6 ± 0.6	2.3 ± 0.9	2.0 ± 0.6	4.3 ± 0.5	4.5 ± 0.2	8.5 ± 1.8
40:6	0.2 ± 0.4	0.2 ± 0.3	1.1 ± 0.2	1.1 ± 0.3	1.9 ± 0.3	2.5 ± 0.7	3.8 ± 1.4	2.7 ± 0.6
40:7	0.3 ± 0.4	0.7 ± 0.6	1.6 ± 0.8	1.5 ± 0.3	2.5 ± 0.5	2.8 ± 0.4	2.3 ± 0.3	1.3 ± 0.7
40:8	16.1 ± 2.5	28.6 ± 6.3	40.1 ± 4.3	73.0 ± 5.4	78.2 ± 6.5	74.8 ± 5.0	42.3 ± 7.8	1.5 ± 0.6
18:2_22:6	*39 %*	*29 %*	*25 %*	*16 %*	*13 %*	*12 %*	*15 %*	*40 %*
18:3_22:5	*1 %*	*1 %*	*1 %*	*1 %*	*0 %*	*0 %*	*1 %*	*3 %*
20:4_20:4	*55 %*	*66 %*	*70 %*	*80 %*	*84 %*	*85 %*	*80 %*	*46 %*
20:5_20:3	*5 %*	*4 %*	*4 %*	*2 %*	*2 %*	*2 %*	*3 %*	*11 %*
40:9	0.2 ± 0.4	0.4 ± 0.4	1.2 ± 0.2	1.6 ± 0.6	1.5 ± 0.5	0.9 ± 0.4	0.0 ± 0.0	0.0 ± 0.0
42:1	0.0 ± 0.0	0.0 ± 0.0	0.0 ± 0.0	0.0 ± 0.0	0.0 ± 0.0	0.2 ± 0.4	0.2 ± 0.4	1.1 ± 0.1
42:2	0.0 ± 0.0	0.0 ± 0.0	0.0 ± 0.0	0.0 ± 0.0	0.0 ± 0.0	0.4 ± 0.3	0.6 ± 0.7	1.0 ± 0.2
42:4	0.0 ± 0.0	0.0 ± 0.0	0.5 ± 0.5	1.0 ± 0.4	1.1 ± 0.4	3.0 ± 0.9	2.0 ± 0.3	2.5 ± 0.5
42:5	0.0 ± 0.0	0.2 ± 0.4	1.1 ± 0.5	1.7 ± 0.3	2.7 ± 0.4	5.2 ± 1.5	2.5 ± 0.5	3.6 ± 1.0
42:6	0.0 ± 0.0	0.0 ± 0.0	0.0 ± 0.0	0.0 ± 0.0	0.0 ± 0.0	0.2 ± 0.4	0.0 ± 0.0	0.5 ± 0.4
42:7	0.0 ± 0.0	0.0 ± 0.0	0.0 ± 0.0	0.0 ± 0.0	0.0 ± 0.0	0.6 ± 0.5	0.6 ± 0.7	0.5 ± 0.4
42:8	0.2 ± 0.3	0.5 ± 0.4	0.5 ± 0.5	1.8 ± 0.7	2.5 ± 0.4	5.8 ± 0.9	4.3 ± 0.8	3.0 ± 0.5
42:9	0.3 ± 0.5	0.6 ± 0.2	1.1 ± 0.0	1.4 ± 0.8	1.3 ± 0.2	1.3 ± 0.9	0.0 ± 0.0	0.0 ± 0.0
42:10	3.2 ± 0.3	5.0 ± 1.6	7.2 ± 0.3	9.0 ± 0.2	8.4 ± 0.2	5.9 ± 0.4	3.4 ± 1.0	0.0 ± 0.0
44:4	0.0 ± 0.0	0.2 ± 0.3	0.9 ± 0.4	1.0 ± 0.2	1.5 ± 0.5	3.2 ± 0.9	2.0 ± 0.4	1.6 ± 0.3
44:5	0.2 ± 0.3	0.4 ± 0.4	0.5 ± 0.5	1.5 ± 0.2	2.2 ± 0.5	3.9 ± 0.9	2.4 ± 0.2	2.0 ± 0.5
44:6	0.0 ± 0.0	0.0 ± 0.0	0.0 ± 0.0	0.0 ± 0.0	0.0 ± 0.0	0.3 ± 0.6	0.9 ± 0.4	0.0 ± 0.0
44:7	0.0 ± 0.0	0.0 ± 0.0	0.0 ± 0.0	0.0 ± 0.0	0.0 ± 0.0	0.0 ± 0.0	0.3 ± 0.5	0.2 ± 0.3
44:8	0.0 ± 0.0	0.0 ± 0.0	0.0 ± 0.0	0.6 ± 0.6	1.2 ± 0.2	3.7 ± 0.7	2.4 ± 1.1	3.3 ± 0.5
**total**	**42.3 ± 14.4**	**64.8 ± 12.6**	**102.5 ± 25.9**	**163.0 ± 20.9**	**189.5 ± 10.4**	**255.2 ± 7.8**	**248.0 ± 14.1**	**296.8 ± 22.5**

SUPPLEMENTAL TABLE S4. Species distribution of the propargylcholine-labeled PC in bEND3 cells upon incubation with LpPC 22:6. Total lipids isolated from cells labeled with 50 µM of tracer for the indicated time were analyzed by MS as multiplexed samples. Labeled lipid species were identified by their specific NL peak and quantified using synthetic pPC 31:1 as internal standard. Molecular species were identified by the FA peaks but omitted from table for clarity. Only the fractions of selected molecular species are shown (*italics*); no *sn*-position information available. Species amounts are shown as pmol per 100,000 cells and represent means ± SD; N=3.

	incubation time							
pPC	10 min	20 min	30 min	60 min	2 h	4 h	8 h	24 h
30:0	0.0 ± 0.0	0.2 ± 0.3	0.2 ± 0.4	0.0 ± 0.0	0.3 ± 0.3	1.0 ± 0.3	1.5 ± 0.2	3.2 ± 0.4
30:1	0.6 ± 0.1	0.9 ± 0.2	0.5 ± 0.1	0.6 ± 0.3	0.7 ± 0.2	0.5 ± 0.2	0.7 ± 0.3	0.5 ± 0.2
32:0	0.3 ± 0.3	0.5 ± 0.4	0.5 ± 0.5	0.6 ± 0.5	1.5 ± 0.1	3.9 ± 0.3	9.5 ± 0.6	21.7 ± 0.9
32:1	2.6 ± 0.8	3.5 ± 1.1	2.4 ± 0.4	3.9 ± 1.4	4.2 ± 1.0	6.4 ± 0.9	12.3 ± 2.1	15.6 ± 2.7
32:2	0.8 ± 0.4	1.6 ± 0.8	1.2 ± 0.2	1.3 ± 0.8	1.8 ± 1.0	1.5 ± 0.5	1.6 ± 0.8	1.1 ± 0.2
32:3	0.0 ± 0.0	0.0 ± 0.0	0.0 ± 0.0	0.0 ± 0.0	0.0 ± 0.0	0.0 ± 0.0	0.0 ± 0.0	0.0 ± 0.0
34:0	0.1 ± 0.1	0.3 ± 0.5	0.4 ± 0.4	0.6 ± 0.6	1.0 ± 0.3	1.0 ± 0.1	2.0 ± 0.4	4.0 ± 0.4
34:1	2.6 ± 0.6	3.1 ± 0.7	3.3 ± 1.5	4.8 ± 1.7	6.3 ± 1.5	14.0 ± 2.1	32.8 ± 2.7	65.5 ± 10.4
34:2	3.2 ± 0.8	4.8 ± 1.3	2.8 ± 1.0	5.6 ± 2.8	4.9 ± 1.5	6.8 ± 1.6	8.7 ± 2.3	10.1 ± 2.7
34:3	0.7 ± 0.1	0.8 ± 0.2	0.6 ± 0.1	1.1 ± 0.4	0.8 ± 0.3	1.4 ± 0.5	1.0 ± 0.3	1.1 ± 0.2
34:4	0.5 ± 0.5	0.6 ± 0.5	0.6 ± 0.1	0.9 ± 0.9	0.6 ± 0.6	0.2 ± 0.3	0.3 ± 0.3	0.5 ± 0.5
36:0	0.0 ± 0.0	0.0 ± 0.0	0.0 ± 0.0	0.0 ± 0.0	0.0 ± 0.0	0.0 ± 0.0	0.1 ± 0.2	0.3 ± 0.3
36:1	0.3 ± 0.3	0.7 ± 0.1	0.5 ± 0.1	0.9 ± 0.3	1.6 ± 0.3	3.7 ± 0.3	8.9 ± 2.4	13.0 ± 2.3
36:2	1.0 ± 0.6	1.3 ± 0.9	0.8 ± 0.9	2.2 ± 0.6	4.1 ± 1.0	8.4 ± 1.6	11.8 ± 2.6	11.6 ± 3.4
36:3	0.8 ± 0.7	1.3 ± 0.2	0.9 ± 0.3	2.3 ± 0.3	2.3 ± 0.6	3.2 ± 0.7	3.6 ± 0.9	4.4 ± 0.6
36:4	3.1 ± 0.4	4.3 ± 1.0	3.3 ± 0.7	5.7 ± 1.7	4.8 ± 1.3	6.1 ± 0.6	4.7 ± 0.8	9.7 ± 2.3
36:5	1.1 ± 0.5	1.6 ± 0.3	1.2 ± 0.2	1.3 ± 0.4	1.1 ± 0.2	1.8 ± 0.6	2.0 ± 0.4	4.2 ± 0.2
36:6	0.6 ± 0.1	0.6 ± 0.6	1.0 ± 0.2	0.8 ± 0.7	1.3 ± 0.1	1.3 ± 0.3	1.4 ± 0.3	1.6 ± 0.3
38:0	0.0 ± 0.0	0.0 ± 0.0	0.0 ± 0.0	0.0 ± 0.0	0.0 ± 0.0	0.0 ± 0.0	0.1 ± 0.2	1.1 ± 0.2
38:1	0.0 ± 0.0	0.0 ± 0.0	0.0 ± 0.0	0.0 ± 0.0	0.5 ± 0.4	1.3 ± 0.7	1.4 ± 0.4	2.1 ± 0.6
38:2	0.0 ± 0.0	0.2 ± 0.4	0.1 ± 0.2	0.6 ± 0.6	0.9 ± 0.3	1.9 ± 0.7	1.7 ± 0.4	1.6 ± 0.6
38:3	0.2 ± 0.3	0.3 ± 0.3	0.2 ± 0.3	0.5 ± 0.5	0.8 ± 0.3	1.6 ± 0.6	2.3 ± 0.4	3.1 ± 0.8
38:4	0.5 ± 0.4	1.1 ± 1.0	1.0 ± 0.9	1.5 ± 1.3	2.4 ± 1.7	4.7 ± 3.7	7.8 ± 6.5	8.2 ± 6.3
38:5	1.3 ± 0.4	1.6 ± 0.2	1.4 ± 0.6	1.9 ± 0.4	3.2 ± 0.8	5.2 ± 0.7	8.5 ± 1.7	10.9 ± 1.3
38:6	2.2 ± 0.4	5.2 ± 0.7	7.6 ± 0.8	10.4 ± 1.4	13.8 ± 0.5	14.9 ± 3.3	17.1 ± 3.1	40.1 ± 6.6
38:7	1.9 ± 0.3	3.7 ± 0.5	6.7 ± 1.4	9.1 ± 1.3	11.7 ± 2.6	7.8 ± 1.4	4.8 ± 1.4	2.8 ± 0.7
38:8	0.0 ± 0.0	0.0 ± 0.0	0.0 ± 0.0	0.0 ± 0.0	0.0 ± 0.0	0.0 ± 0.0	0.1 ± 0.2	0.1 ± 0.1
40:0	0.0 ± 0.0	0.0 ± 0.0	0.0 ± 0.0	0.0 ± 0.0	0.0 ± 0.0	0.0 ± 0.0	0.2 ± 0.3	1.8 ± 0.6
40:1	0.0 ± 0.0	0.0 ± 0.0	0.0 ± 0.0	0.0 ± 0.0	0.8 ± 0.3	1.3 ± 1.0	1.8 ± 0.8	3.0 ± 0.6
40:2	0.0 ± 0.0	0.0 ± 0.0	0.0 ± 0.0	0.6 ± 0.1	0.6 ± 0.6	2.1 ± 0.7	1.9 ± 0.4	2.7 ± 0.8
40:3	0.0 ± 0.0	0.0 ± 0.0	0.0 ± 0.0	0.0 ± 0.0	0.0 ± 0.0	0.4 ± 0.4	0.5 ± 0.5	0.4 ± 0.4
40:4	0.1 ± 0.2	0.0 ± 0.0	0.0 ± 0.0	0.0 ± 0.0	0.2 ± 0.3	0.9 ± 0.2	0.9 ± 0.2	1.5 ± 0.3
40:5	0.0 ± 0.0	0.0 ± 0.0	0.3 ± 0.5	0.9 ± 0.2	0.9 ± 0.1	1.7 ± 0.5	2.3 ± 0.3	2.3 ± 0.9
40:6	1.6 ± 0.5	3.5 ± 1.2	4.4 ± 0.9	8.7 ± 0.9	10.3 ± 1.9	15.2 ± 0.4	26.8 ± 4.2	19.9 ± 2.3
40:7	3.4 ± 1.1	8.0 ± 2.3	14.6 ± 2.4	26.0 ± 1.3	33.3 ± 3.7	30.5 ± 3.8	30.4 ± 7.7	9.6 ± 0.5
40:8	0.4 ± 0.4	1.3 ± 0.6	2.2 ± 0.0	3.8 ± 0.7	4.7 ± 1.0	4.5 ± 0.6	2.6 ± 0.6	0.8 ± 0.2
42:1	0.0 ± 0.0	0.0 ± 0.0	0.0 ± 0.0	0.0 ± 0.0	0.2 ± 0.4	0.3 ± 0.5	0.5 ± 0.9	0.8 ± 1.4
42:2	0.0 ± 0.0	0.0 ± 0.0	0.0 ± 0.0	0.0 ± 0.0	0.3 ± 0.5	1.0 ± 0.9	1.4 ± 1.8	2.0 ± 1.7
42:3	0.0 ± 0.0	0.0 ± 0.0	0.0 ± 0.0	0.0 ± 0.0	0.0 ± 0.0	0.4 ± 0.6	0.0 ± 0.0	0.2 ± 0.3
42:4	0.0 ± 0.0	0.0 ± 0.0	0.0 ± 0.0	0.0 ± 0.0	0.0 ± 0.0	0.8 ± 0.7	0.6 ± 0.5	0.6 ± 0.5
42:5	0.0 ± 0.0	0.0 ± 0.0	0.0 ± 0.0	0.0 ± 0.0	0.2 ± 0.3	1.2 ± 1.1	0.2 ± 0.3	0.7 ± 0.6
42:6	0.0 ± 0.0	0.0 ± 0.0	0.0 ± 0.0	0.0 ± 0.0	0.0 ± 0.0	0.2 ± 0.4	0.3 ± 0.5	1.0 ± 0.8
42:7	0.0 ± 0.0	0.3 ± 0.5	0.2 ± 0.4	0.6 ± 1.1	0.5 ± 0.9	1.1 ± 1.9	0.8 ± 1.3	0.8 ± 1.3
42:9	0.0 ± 0.0	0.6 ± 0.5	1.0 ± 0.6	1.7 ± 0.3	2.3 ± 0.7	1.6 ± 0.2	0.8 ± 0.5	0.1 ± 0.2
42:10	4.7 ± 0.7	9.7 ± 0.7	12.6 ± 1.8	21.0 ± 2.4	25.4 ± 2.2	22.5 ± 3.5	11.7 ± 3.3	0.5 ± 0.5
42:11	0.4 ± 0.3	1.2 ± 0.4	1.9 ± 0.5	3.2 ± 0.4	4.1 ± 0.6	3.6 ± 0.6	2.4 ± 0.8	0.0 ± 0.0
44:4	0.0 ± 0.0	0.0 ± 0.0	0.0 ± 0.0	0.0 ± 0.0	0.0 ± 0.0	0.5 ± 0.5	0.0 ± 0.0	0.2 ± 0.3
44:5	0.0 ± 0.0	0.0 ± 0.0	0.0 ± 0.0	0.0 ± 0.0	0.0 ± 0.0	0.4 ± 0.6	0.2 ± 0.4	0.6 ± 0.5
44:6	0.0 ± 0.0	0.0 ± 0.0	0.0 ± 0.0	0.3 ± 0.5	0.5 ± 0.4	0.3 ± 0.4	0.6 ± 0.6	0.9 ± 0.8
44:7	0.0 ± 0.0	0.0 ± 0.0	0.0 ± 0.0	0.3 ± 0.5	0.2 ± 0.4	0.5 ± 0.9	0.2 ± 0.3	0.4 ± 0.7
44:11	0.2 ± 0.3	0.3 ± 0.5	0.9 ± 0.2	1.9 ± 0.2	1.5 ± 0.3	1.2 ± 0.3	0.4 ± 0.4	0.3 ± 0.4
44:12	8.6 ± 1.0	19.0 ± 4.3	27.3 ± 3.3	49.4 ± 3.9	60.9 ± 3.0	50.6 ± 3.7	41.8 ± 1.1	3.1 ± 0.5
22:6_22:6	*100 %*	*100 %*	*100 %*	*100 %*	*100 %*	*100 %*	*100 %*	*100 %*
**total**	**43.9 ± 5.4**	**82.1 ± 2.2**	**102.7 ± 7.6**	**175.4 ± 14.6**	**217.5 ± 13.8**	**242.0 ± 30.9**	**277.0 ± 28.9**	**292.8 ± 32.9**

SUPPLEMENTAL TABLE S5. Species distribution of the propargylcholine-labeled PC in primary hepatocytes upon incubation with LpPC 16:0. Total lipids isolated from cells labeled with 50 µM of tracer for the indicated time were analyzed by MS. Labeled lipid species were identified by their specific NL peak and quantified using synthetic pPC 31:1 as internal standard. Molecular species were identified by the FA peaks but omitted from table for clarity. Only the fractions of selected molecular species are shown (*italics*); no *sn*-position information available. Species amounts are shown as pmol per 100,000 cells and represent means ± SD; N=3.

	incubation time					
pPC	10 min	20 min	40 min	60 min	2 h	4 h
32:0	0 ± 0	0 ± 0	0.9 ± 1.6	4.8 ± 1.0	12.6 ± 2.6	12.3 ± 3.1
14:0_18:0	*6 %*	*8 %*	*6 %*	*5 %*	*6 %*	*5 %*
16:0_16:0	*94 %*	*92 %*	*94 %*	*95 %*	*94 %*	*95 %*
32:1	0 ± 0	0 ± 0	0 ± 0	0 ± 0	0 ± 0	4.8 ± 0.7
34:0	0 ± 0	0 ± 0	0 ± 0	1.8 ± 1.6	1.2 ± 2.0	1.6 ± 2.8
34:1	0 ± 0	0 ± 0	3.5 ± 0.2	4.3 ± 1.7	15.7 ± 0.3	33.4 ± 0.6
34:2	3.1 ± 2.7	4.0 ± 0.9	9.4 ± 2.4	14.5 ± 3.9	35.3 ± 6.1	74.2 ± 5.1
34:3	0 ± 0	0 ± 0	0.9 ± 1.6	1.5 ± 1.5	1.1 ± 1.8	0 ± 0
36:2	0 ± 0	0 ± 0	0 ± 0	0 ± 0	0 ± 0	5.0 ± 0.6
36:3	0 ± 0	0 ± 0	1.7 ± 2.9	4.1 ± 1.0	6.0 ± 3.5	10.1 ± 1.8
36:4	29.3 ± 11.8	30.6 ± 0.5	66.5 ± 6.3	96.4 ± 17.5	216.9 ± 17.0	289.4 ± 27.6
36:5	5.1 ± 1.1	4.2 ± 1.0	4.5 ± 0.7	8.3 ± 2.8	6.2 ± 0.9	6.0 ± 1.7
38:4	0 ± 0	0 ± 0	0 ± 0	0 ± 0	2.2 ± 1.9	9.9 ± 2.5
38:5	0 ± 0	0.9 ± 1.6	4.6 ± 1.2	7.0 ± 0.7	12.6 ± 1.7	11.1 ± 3.8
38:6	4.2 ± 4.2	3.4 ± 3.9	7.7 ± 0.3	11.2 ± 2.2	18.9 ± 1.8	33.4 ± 6.9
38:7	0 ± 0	0 ± 0	0 ± 0	2.0 ± 1.8	3.5 ± 3.4	0 ± 0
**total pPC**	**41.8 ± 13.0**	**43.1 ± 3.4**	**99.7 ± 14.5**	**155.9 ± 19.6**	**332.0 ± 27.5**	**491.2 ± 43.3**

SUPPLEMENTAL TABLE S6. Species distribution of the propargylcholine-labeled PC in primary hepatocytes upon incubation with LpPC 18:2. Total lipids isolated from cells labeled with 50 µM of tracer for the indicated time were analyzed by MS. Labeled lipid species were identified by their specific NL peak and quantified using synthetic pPC 31:1 as internal standard. Molecular species were identified by the FA peaks but omitted from table for clarity. Only the fractions of selected molecular species are shown (*italics*); no *sn*-position information available. Species amounts are shown as pmol per 100,000 cells and represent means ± SD; N=3.

	incubation time					
pPC	10 min	20 min	40 min	60 min	2 h	4 h
32:2	0 ± 0	0 ± 0	0.9 ± 1.5	1.3 ± 2.3	1.9 ± 1.6	2.3 ± 2.0
34:1	0 ± 0	0 ± 0	0 ± 0	0 ± 0	8.8 ± 1.8	18.2 ± 3.3
34:2	4.9 ± 1.2	6.5 ± 1.6	8.6 ± 1.8	21.5 ± 4.1	55.8 ± 10.2	109.0 ± 28.5
34:3	1.0 ± 1.8	0 ± 0	1.2 ± 2.1	2.9 ± 3.0	3.7 ± 1.0	5.4 ± 0.3
34:4	5.1 ± 1.1	4.2 ± 3.7	5.6 ± 1.0	5.7 ± 1.8	7.5 ± 0.9	4.9 ± 0.6
36:2	0 ± 0	0 ± 0	1.4 ± 2.5	5.9 ± 0.7	14.6 ± 2.1	29.7 ± 8.8
36:3	0 ± 0	0 ± 0	0 ± 0	2.9 ± 2.5	4.7 ± 2.5	8.2 ± 3.9
36:4	18.0 ± 1.8	16.2 ± 2.0	24.6 ± 2.1	37.7 ± 6.0	72.1 ± 8.8	100.3 ± 31.2
16:0_20:4	*75 %*	*72 %*	*69 %*	*63 %*	*67 %*	*69 %*
16:1_20:3	*9 %*	*9 %*	*8 %*	*6 %*	*6 %*	*4 %*
18:2_18:2	*15 %*	*19 %*	*23 %*	*32 %*	*27 %*	*27 %*
36:5	3.1 ± 3.0	5.5 ± 1.3	5.4 ± 1.7	6.5 ± 1.3	9.6 ± 1.6	7.4 ± 2.2
36:6	8.0 ± 1.0	7.8 ± 2.1	7.1 ± 2.7	9.3 ± 1.9	14.0 ± 1.5	11.0 ± 2.1
38:4	0 ± 0	0 ± 0	0 ± 0	1.1 ± 1.8	8.5 ± 3.0	19.2 ± 3.0
38:5	0 ± 0	0 ± 0	0 ± 0	1.3 ± 2.2	3.3 ± 0.5	4.8 ± 1.7
38:6	15.3 ± 1.8	18.4 ± 2.3	35.1 ± 7.2	66.1 ± 8.7	87.9 ± 5.4	89.9 ± 11.0
38:7	0 ± 0	0 ± 0	0 ± 0	0 ± 0	3.5 ± 0.8	2.0 ± 1.7
40:6	0 ± 0	0 ± 0	0 ± 0	0 ± 0	0 ± 0	1.4 ± 2.4
40:8	0 ± 0	0 ± 0	0 ± 0	0 ± 0	2.5 ± 2.1	4.3 ± 1.2
**total pPC**	**55.4 ± 2.8**	**59.5 ± 7.3**	**89.9 ± 3.1**	**162.2 ± 27.9**	**298.5 ± 30.2**	**417.8 ± 99.8**

SUPPLEMENTAL TABLE S7. Species distribution of the propargylcholine-labeled PC in primary hepatocytes upon incubation with LpPC 20:4. Total lipids isolated from cells labeled with 50 µM of tracer for the indicated time were analyzed by MS. Labeled lipid species were identified by their specific NL peak and quantified using synthetic pPC 31:1 as internal standard. Molecular species were identified by the FA peaks but omitted from table for clarity. Only the fractions of selected molecular species are shown (*italics*); no *sn*-position information available. Species amounts are shown as pmol per 100,000 cells and represent means ± SD; N=3.

	incubation time					
pPC	10 min	20 min	40 min	60 min	2 h	4 h
34:1	0 ± 0	0 ± 0	0 ± 0	0 ± 0	12.8 ± 1.8	26.0 ± 0.3
34:2	1.0 ± 1.8	1.1 ± 1.9	2.6 ± 2.3	3.8 ± 4.0	19.9 ± 5.3	41.8 ± 0.4
34:3	0 ± 0	0 ± 0	0 ± 0	0 ± 0	0 ± 0	1.5 ± 2.6
34:4	5.5 ± 1.7	5.9 ± 1.5	5.6 ± 0.6	7.9 ± 2.4	5.1 ± 1.2	9.5 ± 2.3
36:2	0 ± 0	0 ± 0	0 ± 0	0 ± 0	1.2 ± 2.1	6.2 ± 1.2
36:3	0 ± 0	0 ± 0	0 ± 0	0.9 ± 1.6	1.2 ± 2.0	7.5 ± 1.7
36:4	18.2 ± 2.6	25.8 ± 2.7	35.4 ± 5.3	64.8 ± 14.1	113.9 ± 3.4	237.0 ± 16.6
36:5	4.4 ± 0.6	3.4 ± 3.0	7.7 ± 1.7	11.8 ± 3.1	11.4 ± 2.6	11.5 ± 1.6
36:6	7.1 ± 1.3	9.6 ± 1.0	9.7 ± 2.3	13.2 ± 2.7	9.2 ± 3.8	12.0 ± 2.9
38:3	0 ± 0	0 ± 0	0 ± 0	0 ± 0	1.4 ± 2.4	4.5 ± 3.9
38:4	0 ± 0	0.9 ± 1.5	4.5 ± 1.4	8.9 ± 3.0	24.8 ± 5.5	49.5 ± 3.2
38:5	0 ± 0	0 ± 0	1.0 ± 1.7	4.6 ± 1.1	5.4 ± 1.2	10.7 ± 3.9
38:6	2.9 ± 0.6	6.0 ± 2.6	6.8 ± 2.9	13.4 ± 2.7	19.7 ± 2.6	43.2 ± 4.9
38:7	0.7 ± 1.2	0 ± 0	0 ± 0	2.7 ± 2.4	1.2 ± 2.1	0 ± 0
40:8	3.8 ± 1.2	9.6 ± 2.4	22.3 ± 4.6	40.4 ± 7.0	42.0 ± 11.1	34.6 ± 6.8
18:2_22:6	*17 %*	*8 %*	*5 %*	*3 %*	*2 %*	*3 %*
20:4_20:4	*83 %*	*92 %*	*95 %*	*97 %*	*98 %*	*97 %*
**total pPC**	**43.5 ± 1.7**	**62.4 ± 7.6**	**95.5 ± 13.1**	**172.5 ± 39.3**	**269.1 ± 22.1**	**495.5 ± 16.1**

SUPPLEMENTAL TABLE S8. Species distribution of the propargylcholine-labeled PC in primary hepatocytes upon incubation with LpPC 22:6. Total lipids isolated from cells labeled with 50 µM of tracer for the indicated time were analyzed by MS. Labeled lipid species were identified by their specific NL peak and quantified using synthetic pPC 31:1 as internal standard. Molecular species were identified by the FA peaks but omitted from table for clarity. Only the fractions of selected molecular species are shown (*italics*); no *sn*-position information available. Species amounts are shown as pmol per 100,000 cells and represent means ± SD; N=3.

	incubation time					
pPC	10 min	20 min	40 min	60 min	2 h	4 h
32:0	0 ± 0	0 ± 0	0 ± 0	0 ± 0	0 ± 0	0.7 ± 1.2
32:1	0 ± 0	0 ± 0	0 ± 0	0.8 ± 1.4	0 ± 0	1.7 ± 1.5
34:1	0 ± 0	0 ± 0	0 ± 0	3.5 ± 1.2	12.6 ± 1.7	31.8 ± 4.0
34:2	2.7 ± 0.4	2.5 ± 0.8	3.8 ± 1.0	6.4 ± 0.8	21.4 ± 6.1	51.5 ± 4.5
34:3	0.3 ± 0.6	0 ± 0	0 ± 0	0.9 ± 1.6	3.0 ± 1.6	2.6 ± 0.3
34:4	4.7 ± 2.3	4.0 ± 1.9	6.2 ± 1.6	6.7 ± 1.0	7.4 ± 1.7	5.4 ± 1.1
36:1	0 ± 0	0 ± 0	0 ± 0	0 ± 0	0 ± 0	1.0 ± 1.7
36:2	0 ± 0	0 ± 0	0 ± 0	0 ± 0	2.0 ± 1.7	7.7 ± 0.6
36:3	0 ± 0	0 ± 0	0 ± 0	1.7 ± 1.5	4.5 ± 0.8	6.6 ± 1.6
36:4	15.7 ± 2.0	15.0 ± 2.2	22.8 ± 3.7	36.9 ± 2.7	58.8 ± 4.9	120.9 ± 10.8
36:5	3.7 ± 0.3	3.9 ± 1.5	4.5 ± 0.4	7.0 ± 2.4	9.6 ± 1.8	10.6 ± 0.8
36:6	7.5 ± 2.8	7.8 ± 1.1	8.8 ± 2.4	12.8 ± 2.9	17.4 ± 3.8	14.6 ± 2.8
38:4	0 ± 0	0 ± 0	0 ± 0	4.2 ± 1.0	10.0 ± 1.5	31.2 ± 2.0
38:5	0 ± 0	0.5 ± 0.8	0 ± 0	0 ± 0	1.1 ± 2.0	5.2 ± 1.1
38:6	4.6 ± 0.8	7.2 ± 0.8	20.0 ± 2.8	37.3 ± 4.8	117.3 ± 9.2	273.7 ± 3.9
38:7	1.6 ± 0.5	2.2 ± 0.5	4.5 ± 0.7	4.4 ± 1.5	7.5 ± 0.5	8.4 ± 2.2
40:6	0 ± 0	0.6 ± 1.1	2.1 ± 2.1	5.0 ± 2.0	11.4 ± 3.3	30.9 ± 5.1
40:7	0 ± 0	0 ± 0	0 ± 0	0 ± 0	0 ± 0	1.0 ± 1.7
40:8	0.4 ± 0.7	1.6 ± 1.5	2.4 ± 2.2	6.9 ± 0.7	11.7 ± 2.8	17.5 ± 2.3
42:10	3.7 ± 0.4	6.2 ± 1.1	19.3 ± 4.6	40.1 ± 5.1	64.4 ± 7.4	55.4 ± 5.1
44:12	0 ± 0	0.5 ± 0.9	0 ± 0	6.0 ± 2.8	11.0 ± 1.2	9.7 ± 0.9
22:6_22:6		*100 %*		*100 %*	*100 %*	*100 %*
**total pPC**	**45.0 ± 7.9**	**52.1 ± 9.1**	**94.3 ± 12.4**	**180.5 ± 15.0**	**371.2 ± 33.8**	**688.0 ± 23.5**

SUPPLEMENTAL TABLE S9. Species distribution of the propargylcholine-labeled PC in bEND3 cells upon pulse-chase-incubation using LpPC 16:0. Cells were incubated with 50 µM of LpPC 16:0 in the presence of 50 µM FA 16:0[13C16] for 30 min and chased using 50 µM FA18:1[13C18] for the indicated times. Total cellular lipids were analyzed by MS. Labeled lipid species were identified by their specific NL peak and quantified using synthetic pPC 31:1 or LpPC 14:0 as internal standard. Molecular species were identified by the FA peaks and recorded but omitted from table for clarity. Lipid amounts are shown as pmol per 100,000 cells and represent means ± SD; N=3. Symmetric pPC species carrying the istotope-labeled side chain (red) or the unlabeled homologue (magenta), and asymmetric pPC containing 13C-labeled oleate provided during chase (green) are highlighted and the color code refers to Fig. 3.

	chase time upon 30 min pulse incubation							
pPC	0 min	10 min	20 min	40 min	1 h	2 h	4 h	24 h
32:0	8.3 ± 1.1	7.2 ± 0.7	9.7 ± 0.9	4.7 ± 2.9	5.8 ± 2.3	1.6 ± 1.5	0.8 ± 1.5	0.0 ± 0.0
32:1	6.1 ± 2.2	3.8 ± 0.5	7.3 ± 1.5	5.7 ± 1.6	4.6 ± 1.3	6.3 ± 2.2	5.6 ± 1.1	1.7 ± 2.9
34:0	1.1 ± 1.9	0.0 ± 0.0	1.8 ± 1.6	0.6 ± 1.0	0.0 ± 0.0	0.0 ± 0.0	0.0 ± 0.0	0.0 ± 0.0
34:1	13.2 ± 1.9	11.5 ± 2.3	17.0 ± 0.3	12.4 ± 2.6	13.0 ± 4.2	11.2 ± 1.6	13.2 ± 2.1	6.3 ± 2.3
34:2	6.4 ± 1.9	3.9 ± 1.6	6.4 ± 0.6	6.4 ± 1.1	6.1 ± 0.8	5.9 ± 1.5	4.7 ± 1.7	3.3 ± 2.9
34:3	0.0 ± 0.0	0.0 ± 0.0	0.0 ± 0.0	0.7 ± 1.2	0.0 ± 0.0	0.0 ± 0.0	0.0 ± 0.0	0.0 ± 0.0
36:1	0.9 ± 1.5	0.0 ± 0.0	1.6 ± 1.4	1.7 ± 1.5	0.6 ± 1.1	1.5 ± 1.3	0.8 ± 1.4	0.0 ± 0.0
36:2	2.3 ± 2.2	0.7 ± 1.3	1.5 ± 1.3	1.1 ± 1.0	1.9 ± 1.7	2.4 ± 0.8	2.5 ± 2.5	1.5 ± 2.7
36:3	2.1 ± 1.9	0.8 ± 1.3	2.6 ± 0.2	0.6 ± 1.0	0.0 ± 0.0	0.7 ± 1.1	0.0 ± 0.0	0.0 ± 0.0
36:4	22.1 ± 8.0	16.9 ± 1.6	20.2 ± 6.2	14.2 ± 0.9	18.1 ± 5.3	8.0 ± 2.4	9.8 ± 1.4	3.8 ± 0.8
36:5	1.1 ± 1.9	4.2 ± 0.1	1.2 ± 2.0	1.7 ± 1.5	3.0 ± 2.6	1.9 ± 1.8	2.5 ± 2.2	0.0 ± 0.0
38:4	0.9 ± 1.5	0.8 ± 1.5	1.6 ± 1.4	0.7 ± 1.3	2.5 ± 2.2	0.7 ± 1.2	0.0 ± 0.0	0.0 ± 0.0
38:5	4.1 ± 1.0	4.7 ± 1.8	4.5 ± 1.5	3.0 ± 2.7	5.0 ± 1.2	4.0 ± 1.1	2.4 ± 2.2	2.4 ± 2.0
38:6	11.2 ± 3.3	7.6 ± 2.6	6.2 ± 1.8	2.6 ± 2.4	2.7 ± 2.6	2.5 ± 0.5	1.4 ± 2.4	0.0 ± 0.0
**total**	**79.7 ± 26.6**	**62.2 ± 3.3**	**81.7 ± 4.6**	**56.1 ± 9.2**	**63.3 ± 15.0**	**46.8 ± 8.8**	**43.7 ± 14.1**	**18.9 ± 6.5**
32:0[13C16]	27.8 ± 8.1	19.9 ± 2.7	19.0 ± 2.3	7.9 ± 0.4	9.9 ± 2.4	3.2 ± 0.1	2.0 ± 2.0	0.0 ± 0.0
34:1[13C18]	0.0 ± 0.0	6.7 ± 2.4	8.6 ± 1.4	13.5 ± 2.5	15.6 ± 2.2	16.0 ± 4.3	18.6 ± 0.6	9.8 ± 1.8
34:2[13C18]	0.0 ± 0.0	0.9 ± 1.6	2.9 ± 0.6	2.5 ± 0.6	2.9 ± 0.6	3.9 ± 1.0	2.4 ± 2.2	0.8 ± 1.5
36:2[13C18]	0.0 ± 0.0	0.0 ± 0.0	0.0 ± 0.0	0.0 ± 0.0	0.0 ± 0.0	0.0 ± 0.0	0.0 ± 0.0	2.7 ± 2.3
**total[13C18]**	**0.0 ± 0.0**	**7.6 ± 2.6**	**11.5 ± 1.7**	**16.0 ± 2.4**	**18.6 ± 2.3**	**19.9 ± 5.2**	**21.0 ± 1.6**	**13.3 ± 2.4**

SUPPLEMENTAL TABLE S10. Species distribution of the propargylcholine-labeled PC in bEND3 cells upon pulse-chase-incubation using LpPC 18:2. Cells were incubated with 50 µM of LpPC 18:2 in the presence of 50 µM FA 18:2[13C18] for 30 min and chased using 50 µM FA18:1[13C18] for the indicated times. Total cellular lipids were analyzed by MS. Labeled lipid species were identified by their specific NL peak and quantified using synthetic pPC 31:1 or LpPC 14:0 as internal standard. Molecular species were identified by the FA peaks and recorded but omitted from table for clarity. Lipid amounts are shown as pmol per 100,000 cells and represent means ± SD; N=3. Symmetric pPC species carrying the istotope-labeled side chain (red) or the unlabeled homologue (magenta), and asymmetric pPC containing 13C-labeled oleate provided during chase (green) are highlighted and the color code refers to Fig. 4.

	chase time upon 30 min pulse incubation							
pPC	0 min	10 min	20 min	40 min	1 h	2 h	4 h	24 h
32:1	1.7 ± 0.5	1.6 ± 0.3	2.2 ± 0.2	1.3 ± 1.3	1.1 ± 1.0	1.6 ± 1.4	2.7 ± 0.5	0.6 ± 1.0
32:2	0.7 ± 0.6	0.0 ± 0.0	0.3 ± 0.5	0.0 ± 0.0	0.0 ± 0.0	0.0 ± 0.0	0.0 ± 0.0	0.0 ± 0.0
34:0	0.0 ± 0.0	0.0 ± 0.0	0.4 ± 0.6	0.5 ± 0.9	0.0 ± 0.0	0.0 ± 0.0	0.0 ± 0.0	0.0 ± 0.0
34:1	1.1 ± 0.1	1.6 ± 0.2	1.6 ± 0.5	1.1 ± 1.0	2.7 ± 0.1	2.9 ± 0.3	3.2 ± 0.7	4.7 ± 2.1
34:2	3.6 ± 0.6	4.8 ± 1.0	5.0 ± 1.5	4.3 ± 0.9	5.4 ± 0.6	5.5 ± 1.4	3.7 ± 0.6	2.5 ± 0.7
34:3	2.4 ± 1.0	3.0 ± 0.6	3.7 ± 0.7	2.5 ± 0.5	1.7 ± 1.5	2.6 ± 0.3	0.7 ± 1.3	0.0 ± 0.0
34:4	0.0 ± 0.0	0.5 ± 0.8	0.0 ± 0.0	0.0 ± 0.0	0.0 ± 0.0	0.0 ± 0.0	0.0 ± 0.0	0.0 ± 0.0
36:2	3.9 ± 0.7	3.5 ± 1.3	4.5 ± 1.0	4.5 ± 1.4	4.6 ± 1.9	6.1 ± 3.2	4.9 ± 0.1	1.7 ± 1.5
36:3	2.2 ± 0.3	1.5 ± 1.5	1.2 ± 1.1	1.2 ± 2.1	0.6 ± 1.1	0.4 ± 0.7	0.4 ± 0.8	0.0 ± 0.0
36:4	19.9 ± 2.1	17.9 ± 4.7	17.7 ± 5.1	14.9 ± 3.8	14.0 ± 3.8	13.5 ± 3.0	5.5 ± 1.7	4.6 ± 1.9
36:5	0.0 ± 0.0	0.5 ± 0.9	0.0 ± 0.0	0.5 ± 0.9	0.0 ± 0.0	0.0 ± 0.0	1.1 ± 0.9	0.5 ± 1.0
38:3	0.0 ± 0.0	0.0 ± 0.0	0.0 ± 0.0	0.0 ± 0.0	1.0 ± 1.8	0.0 ± 0.0	0.0 ± 0.0	0.6 ± 1.1
38:4	0.0 ± 0.0	0.8 ± 0.7	0.5 ± 0.8	0.0 ± 0.0	1.0 ± 0.9	1.4 ± 0.1	1.4 ± 0.5	1.3 ± 1.2
38:5	1.3 ± 0.2	0.5 ± 0.8	0.3 ± 0.4	0.0 ± 0.0	0.0 ± 0.0	0.0 ± 0.0	0.0 ± 0.0	0.0 ± 0.0
38:6	14.2 ± 2.3	12.5 ± 2.0	10.8 ± 0.4	7.3 ± 0.8	6.7 ± 1.5	6.6 ± 1.6	2.0 ± 1.7	0.0 ± 0.0
38:7	1.5 ± 0.4	1.0 ± 0.9	0.5 ± 0.9	0.0 ± 0.0	0.0 ± 0.0	0.0 ± 0.0	0.0 ± 0.0	0.0 ± 0.0
40:7	1.0 ± 0.9	1.7 ± 0.4	0.5 ± 0.9	0.0 ± 0.0	0.0 ± 0.0	0.0 ± 0.0	0.0 ± 0.0	0.0 ± 0.0
40:8	4.8 ± 1.5	3.5 ± 0.6	1.8 ± 1.8	0.0 ± 0.0	0.0 ± 0.0	0.0 ± 0.0	0.0 ± 0.0	0.0 ± 0.0
**total**	**58.3 ± 4.5**	**54.8 ± 11.7**	**51.1 ± 11.3**	**38.1 ± 4.6**	**38.9 ± 7.5**	**40.6 ± 10.0**	**25.7 ± 4.0**	**16.5 ± 5.4**
36:4[13C18:2]	37.2 ± 5.1	36.9 ± 6.3	40.9 ± 8.3	24.1 ± 4.7	25.2 ± 0.5	18.6 ± 3.7	10.7 ± 0.8	0.0 ± 0.0
34:1[13C18:1]	0.0 ± 0.0	0.0 ± 0.0	0.0 ± 0.0	0.0 ± 0.0	0.0 ± 0.0	0.4 ± 0.7	2.6 ± 0.5	5.7 ± 0.9
34:2[13C18:1]	0.0 ± 0.0	0.0 ± 0.0	0.0 ± 0.0	0.0 ± 0.0	0.0 ± 0.0	0.5 ± 0.8	1.1 ± 0.9	0.8 ± 1.4
36:2[13C18:1]	0.0 ± 0.0	0.0 ± 0.0	0.0 ± 0.0	0.0 ± 0.0	0.5 ± 0.8	0.4 ± 0.7	2.3 ± 0.1	3.3 ± 1.7
36:3[13C18:1]	0.0 ± 0.0	0.0 ± 0.0	0.0 ± 0.0	0.0 ± 0.0	0.0 ± 0.0	0.0 ± 0.0	0.0 ± 0.0	0.0 ± 0.0
**total[13C18:1]**	**0.0 ± 0.0**	**0.0 ± 0.0**	**0.0 ± 0.0**	**0.0 ± 0.0**	**0.5 ± 0.8**	**1.2 ± 2.2**	**6.0 ± 1.2**	**9.9 ± 1.3**

SUPPLEMENTAL TABLE S11. Species distribution of the propargylcholine-labeled PC in bEND3 cells upon pulse-chase-incubation using LpPC 20:4. Cells were incubated with 50 µM of LpPC 20:4 in the presence of 50 µM FA 20:4[D8] for 30 min and chased using 50 µM FA18:1[13C18] for the indicated times. Total cellular lipids were analyzed by MS. Labeled lipid species were identified by their specific NL peak and quantified using synthetic pPC 31:1 as internal standard. Molecular species were identified by the FA peaks and recorded but omitted from table for clarity. Lipid amounts are shown as pmol per 100,000 cells and represent means ± SD; N=3. Symmetric pPC species carrying the istotope-labeled side chain (red) or the unlabeled homologue (magenta), and asymmetric pPC containing 13C-labeled oleate provided during chase (green) are highlighted and the color code refers to Fig. 3.

	chase time upon 30 min pulse incubation							
pPC	0 min	10 min	20 min	40 min	1 h	2 h	4 h	24 h
32:0	0.0 ± 0.0	0.0 ± 0.0	0.0 ± 0.0	0.0 ± 0.0	0.0 ± 0.0	0.0 ± 0.0	0.0 ± 0.0	1.0 ± 0.9
32:1	0.6 ± 1.0	0.9 ± 0.8	2.2 ± 0.6	0.6 ± 1.1	0.6 ± 1.0	2.4 ± 0.3	1.5 ± 1.3	0.6 ± 1.1
34:0	0.0 ± 0.0	0.0 ± 0.0	0.0 ± 0.0	0.0 ± 0.0	0.0 ± 0.0	0.0 ± 0.0	0.0 ± 0.0	0.5 ± 0.8
34:1	0.5 ± 0.9	1.3 ± 1.2	2.5 ± 0.7	0.9 ± 1.6	3.1 ± 2.8	4.0 ± 0.5	2.9 ± 0.9	5.8 ± 2.0
34:2	1.6 ± 1.5	2.0 ± 0.5	2.7 ± 0.7	2.6 ± 0.5	1.7 ± 1.5	3.2 ± 1.3	2.7 ± 0.2	1.3 ± 1.2
34:3	0.0 ± 0.0	0.4 ± 0.7	0.0 ± 0.0	0.0 ± 0.0	0.0 ± 0.0	0.0 ± 0.0	0.0 ± 0.0	0.0 ± 0.0
34:4	0.3 ± 0.6	0.0 ± 0.0	0.0 ± 0.0	0.0 ± 0.0	0.0 ± 0.0	0.0 ± 0.0	0.0 ± 0.0	0.0 ± 0.0
36:1	0.0 ± 0.0	0.0 ± 0.0	0.0 ± 0.0	0.0 ± 0.0	0.0 ± 0.0	0.0 ± 0.0	0.0 ± 0.0	0.5 ± 0.9
36:2	0.0 ± 0.0	0.3 ± 0.6	0.0 ± 0.0	0.0 ± 0.0	0.4 ± 0.7	0.0 ± 0.0	0.0 ± 0.0	0.4 ± 0.8
36:3	0.0 ± 0.0	0.8 ± 0.7	0.0 ± 0.0	0.0 ± 0.0	0.5 ± 0.8	0.0 ± 0.0	0.7 ± 1.1	0.9 ± 1.6
36:4	4.0 ± 0.6	4.3 ± 2.2	4.2 ± 1.2	4.7 ± 0.6	4.2 ± 0.5	3.6 ± 0.7	3.5 ± 0.9	5.7 ± 1.5
36:5	3.6 ± 1.3	2.5 ± 0.5	4.1 ± 2.1	3.2 ± 1.1	2.6 ± 1.0	1.0 ± 0.9	0.0 ± 0.0	0.4 ± 0.7
38:4	4.0 ± 1.1	3.8 ± 1.4	5.0 ± 0.9	2.7 ± 2.4	5.4 ± 0.5	4.9 ± 0.2	3.4 ± 0.8	2.6 ± 1.1
38:5	4.5 ± 1.8	4.9 ± 1.9	5.6 ± 1.0	5.2 ± 2.3	4.0 ± 1.0	3.3 ± 0.3	1.4 ± 1.2	0.7 ± 1.3
38:6	1.9 ± 1.6	1.8 ± 0.7	0.7 ± 1.3	1.5 ± 1.3	1.1 ± 1.0	0.5 ± 0.9	1.8 ± 1.6	0.0 ± 0.0
40:5	0.7 ± 1.2	0.2 ± 0.4	0.6 ± 1.0	0.0 ± 0.0	0.0 ± 0.0	0.0 ± 0.0	0.0 ± 0.0	0.0 ± 0.0
40:6	0.0 ± 0.0	0.4 ± 0.7	0.0 ± 0.0	0.0 ± 0.0	0.0 ± 0.0	0.0 ± 0.0	0.0 ± 0.0	0.0 ± 0.0
40:7	1.9 ± 1.7	1.8 ± 0.8	1.0 ± 1.8	0.0 ± 0.0	0.0 ± 0.0	0.0 ± 0.0	0.0 ± 0.0	0.0 ± 0.0
40:8	17.8 ± 3.9	14.2 ± 3.5	15.6 ± 3.8	12.4 ± 3.1	6.9 ± 2.6	2.1 ± 1.9	0.0 ± 0.0	0.0 ± 0.0
42:9	0.4 ± 0.7	0.0 ± 0.0	0.0 ± 0.0	0.0 ± 0.0	0.0 ± 0.0	0.0 ± 0.0	0.0 ± 0.0	0.0 ± 0.0
42:10	6.3 ± 2.2	3.8 ± 1.1	3.9 ± 1.6	1.7 ± 1.5	0.0 ± 0.0	0.0 ± 0.0	0.0 ± 0.0	0.0 ± 0.0
**total**	**48.0 ± 8.3**	**43.4 ± 5.7**	**48.1 ± 11.2**	**35.5 ± 12.8**	**30.3 ± 10.4**	**24.9 ± 4.0**	**17.9 ± 3.7**	**20.6 ± 9.7**
40:8[D8]	10.3 ± 3.6	7.7 ± 2.3	11.2 ± 2.6	6.4 ± 1.5	4.4 ± 1.0	1.6 ± 1.5	0.0 ± 0.0	0.0 ± 0.0
34:1[13C18]	0.0 ± 0.0	0.0 ± 0.0	0.0 ± 0.0	0.0 ± 0.0	0.0 ± 0.0	0.7 ± 1.1	0.6 ± 1.0	6.9 ± 1.6
34:2[13C18]	0.0 ± 0.0	0.0 ± 0.0	0.0 ± 0.0	0.0 ± 0.0	0.0 ± 0.0	0.0 ± 0.0	0.0 ± 0.0	1.2 ± 1.0
36:2[13C18]	0.0 ± 0.0	0.0 ± 0.0	0.0 ± 0.0	0.0 ± 0.0	0.0 ± 0.0	0.5 ± 0.8	0.0 ± 0.0	1.4 ± 1.3
38:5[13C18]	0.0 ± 0.0	0.0 ± 0.0	0.8 ± 1.4	0.7 ± 1.3	0.0 ± 0.0	1.0 ± 1.7	0.0 ± 0.0	0.7 ± 1.2
**total[13C18]**	**0.0 ± 0.0**	**0.0 ± 0.0**	**0.8 ± 1.4**	**0.7 ± 1.3**	**0.0 ± 0.0**	**2.1 ± 1.9**	**0.6 ± 1.0**	**10.2 ± 4.5**

SUPPLEMENTAL TABLE S12. Species distribution of the propargylcholine-labeled PC in bEND3 cells upon pulse-chase-incubation using LpPC 22:6. Cells were incubated with 50 µM of LpPC 22:6 in the presence of 50 µM FA 22:6[D5] for 30 min and chased using 50 µM FA18:1[13C18] for the indicated times. Total cellular lipids were analyzed by MS. Labeled lipid species were identified by their specific NL peak and quantified using synthetic pPC 31:1 as internal standard. Molecular species were identified by the FA peaks and recorded but omitted from table for clarity. Lipid amounts are shown as pmol per 100,000 cells and represent means ± SD; N=3. Symmetric pPC species carrying the istotope-labeled side chain (red) or the unlabeled homologue (magenta), and asymmetric pPC containing 13C-labeled oleate provided during chase (green) are highlighted and the color code refers to Fig. 3.

	chase time upon 30 min pulse incubation							
pPC	0 min	10 min	20 min	40 min	1 h	2 h	4 h	24 h
30:0	0.0 ± 0.1	0.1 ± 0.1	0.0 ± 0.0	0.1 ± 0.1	0.1 ± 0.1	0.1 ± 0.1	0.1 ± 0.1	0.0 ± 0.0
30:1	0.2 ± 0.1	0.2 ± 0.1	0.2 ± 0.0	0.0 ± 0.1	0.2 ± 0.0	0.2 ± 0.0	0.1 ± 0.2	0.0 ± 0.0
32:0	0.2 ± 0.1	0.3 ± 0.1	0.3 ± 0.0	0.2 ± 0.2	0.3 ± 0.0	0.5 ± 0.2	0.4 ± 0.1	0.7 ± 0.1
32:1	0.7 ± 0.1	0.8 ± 0.2	0.8 ± 0.1	0.9 ± 0.1	1.1 ± 0.3	1.2 ± 0.2	1.1 ± 0.2	0.8 ± 0.1
32:2	0.2 ± 0.0	0.2 ± 0.0	0.1 ± 0.1	0.1 ± 0.1	0.2 ± 0.0	0.2 ± 0.1	0.2 ± 0.1	0.0 ± 0.0
34:0	0.0 ± 0.0	0.1 ± 0.1	0.1 ± 0.1	0.0 ± 0.0	0.0 ± 0.1	0.1 ± 0.1	0.1 ± 0.1	0.1 ± 0.1
34:1	0.7 ± 0.1	0.8 ± 0.2	0.9 ± 0.0	1.2 ± 0.2	1.5 ± 0.3	2.1 ± 0.2	2.2 ± 0.2	1.8 ± 0.3
34:2	1.1 ± 0.2	0.9 ± 0.1	1.0 ± 0.2	0.9 ± 0.1	1.1 ± 0.1	1.3 ± 0.2	1.2 ± 0.2	0.6 ± 0.2
34:3	0.1 ± 0.1	0.2 ± 0.0	0.1 ± 0.1	0.1 ± 0.1	0.0 ± 0.1	0.1 ± 0.1	0.0 ± 0.0	0.0 ± 0.0
34:4	0.2 ± 0.1	0.2 ± 0.1	0.2 ± 0.2	0.0 ± 0.0	0.2 ± 0.2	0.1 ± 0.1	0.1 ± 0.1	0.0 ± 0.0
36:1	0.0 ± 0.0	0.0 ± 0.0	0.0 ± 0.0	0.1 ± 0.1	0.0 ± 0.0	0.0 ± 0.0	0.1 ± 0.1	0.0 ± 0.0
36:2	0.2 ± 0.0	0.3 ± 0.1	0.4 ± 0.1	0.3 ± 0.1	0.6 ± 0.1	0.7 ± 0.2	0.7 ± 0.1	0.5 ± 0.1
36:3	0.4 ± 0.1	0.3 ± 0.1	0.3 ± 0.0	0.3 ± 0.1	0.4 ± 0.1	0.5 ± 0.1	0.4 ± 0.4	0.4 ± 0.1
36:4	1.0 ± 0.1	0.9 ± 0.2	1.1 ± 0.2	0.8 ± 0.1	0.8 ± 0.1	1.1 ± 0.0	1.0 ± 0.2	0.9 ± 0.3
36:5	0.3 ± 0.0	0.3 ± 0.1	0.3 ± 0.1	0.1 ± 0.1	0.2 ± 0.1	0.2 ± 0.0	0.1 ± 0.1	0.0 ± 0.0
36:6	0.4 ± 0.1	0.6 ± 0.1	0.4 ± 0.0	0.2 ± 0.2	0.2 ± 0.1	0.1 ± 0.1	0.0 ± 0.0	0.0 ± 0.1
38:3	0.0 ± 0.0	0.0 ± 0.0	0.0 ± 0.0	0.0 ± 0.0	0.0 ± 0.0	0.1 ± 0.1	0.0 ± 0.0	0.0 ± 0.0
38:4	0.5 ± 0.1	0.5 ± 0.0	0.7 ± 0.2	0.8 ± 0.2	0.9 ± 0.1	1.1 ± 0.2	0.7 ± 0.2	0.5 ± 0.1
38:5	0.5 ± 0.2	0.4 ± 0.1	0.5 ± 0.1	0.7 ± 0.2	0.7 ± 0.1	0.8 ± 0.1	0.8 ± 0.1	0.5 ± 0.1
38:6	3.9 ± 0.6	3.9 ± 0.3	3.4 ± 0.2	2.7 ± 0.2	3.0 ± 0.7	1.7 ± 0.3	0.7 ± 0.6	0.3 ± 0.1
38:7	2.7 ± 0.2	2.7 ± 0.4	2.8 ± 0.4	2.1 ± 0.2	2.2 ± 0.2	1.0 ± 0.1	0.2 ± 0.2	0.0 ± 0.0
40:4	0.0 ± 0.0	0.0 ± 0.0	0.0 ± 0.0	0.0 ± 0.0	0.0 ± 0.0	0.1 ± 0.1	0.1 ± 0.1	0.0 ± 0.0
40:5	0.2 ± 0.1	0.3 ± 0.0	0.2 ± 0.2	0.1 ± 0.1	0.2 ± 0.1	0.0 ± 0.0	0.0 ± 0.0	0.0 ± 0.0
40:6	2.2 ± 0.4	2.3 ± 0.3	2.4 ± 0.2	1.6 ± 0.1	1.5 ± 0.3	1.0 ± 0.2	0.4 ± 0.4	0.0 ± 0.0
40:7	8.3 ± 0.5	8.6 ± 1.0	8.1 ± 1.3	6.6 ± 0.5	5.9 ± 0.2	3.2 ± 0.0	1.0 ± 0.3	0.0 ± 0.0
40:8	1.4 ± 0.1	1.5 ± 0.1	1.4 ± 0.3	1.1 ± 0.2	1.0 ± 0.2	0.5 ± 0.1	0.0 ± 0.0	0.0 ± 0.0
42:7	0.3 ± 0.1	0.3 ± 0.0	0.2 ± 0.1	0.2 ± 0.2	0.1 ± 0.1	0.0 ± 0.0	0.0 ± 0.0	0.0 ± 0.0
42:9	0.8 ± 0.2	0.8 ± 0.2	0.5 ± 0.0	0.6 ± 0.1	0.5 ± 0.2	0.2 ± 0.2	0.0 ± 0.0	0.0 ± 0.0
42:10	7.1 ± 1.2	7.4 ± 0.4	5.8 ± 0.9	4.9 ± 0.5	4.2 ± 0.4	1.8 ± 0.2	0.4 ± 0.1	0.0 ± 0.0
42:11	0.7 ± 0.1	0.9 ± 0.1	0.7 ± 0.1	0.5 ± 0.1	0.4 ± 0.1	0.1 ± 0.2	0.0 ± 0.0	0.0 ± 0.0
44:10	0.1 ± 0.1	0.1 ± 0.1	0.0 ± 0.0	0.1 ± 0.2	0.1 ± 0.1	0.0 ± 0.0	0.0 ± 0.0	0.0 ± 0.0
44:11	2.5 ± 0.3	2.4 ± 0.3	1.9 ± 0.6	1.0 ± 0.1	0.8 ± 0.1	0.1 ± 0.1	0.0 ± 0.0	0.0 ± 0.0
44:12	18.1 ± 1.7	15.8 ± 2.2	10.8 ± 1.2	6.4 ± 0.6	4.9 ± 0.8	1.4 ± 0.3	0.2 ± 0.2	0.0 ± 0.0
**total**	**54.9 ± 3.7**	**54.0 ± 5.1**	**45.5 ± 5.8**	**34.8 ± 0.7**	**33.2 ± 3.3**	**21.6 ± 1.6**	**12.1 ± 3.2**	**7.0 ± 1.4**
44:12[D5]	6.0 ± 0.6	4.6 ± 0.7	4.0 ± 0.8	2.5 ± 0.4	2.0 ± 0.3	0.0 ± 0.0	0.0 ± 0.0	0.0 ± 0.0
34:1[13C18]	0.0 ± 0.0	0.0 ± 0.1	0.0 ± 0.0	0.3 ± 0.0	0.4 ± 0.0	1.1 ± 0.2	1.4 ± 0.4	3.5 ± 0.3
34:2[13C18]	0.0 ± 0.0	0.0 ± 0.0	0.0 ± 0.1	0.1 ± 0.2	0.3 ± 0.1	0.4 ± 0.1	0.6 ± 0.1	0.4 ± 0.1
36:1[13C18]	0.0 ± 0.0	0.0 ± 0.0	0.1 ± 0.1	0.0 ± 0.0	0.2 ± 0.2	0.4 ± 0.0	0.2 ± 0.2	0.2 ± 0.1
36:2[13C18]	0.0 ± 0.0	0.0 ± 0.0	0.0 ± 0.1	0.2 ± 0.0	0.4 ± 0.1	0.6 ± 0.1	0.5 ± 0.5	0.7 ± 0.1
36:3[13C18]	0.0 ± 0.0	0.0 ± 0.0	0.0 ± 0.0	0.0 ± 0.0	0.0 ± 0.0	0.1 ± 0.1	0.0 ± 0.0	0.1 ± 0.1
38:5[13C18]	0.0 ± 0.0	0.0 ± 0.0	0.0 ± 0.0	0.1 ± 0.1	0.3 ± 0.1	0.4 ± 0.2	0.3 ± 0.2	0.3 ± 0.0
40:7[13C18]	0.0 ± 0.0	1.1 ± 0.1	1.7 ± 0.1	2.0 ± 0.3	2.1 ± 0.1	1.5 ± 0.2	0.4 ± 0.4	0.1 ± 0.1
**total[13C18]**	**0.0 ± 0.0**	**1.2 ± 0.1**	**1.9 ± 0.1**	**2.7 ± 0.3**	**3.7 ± 0.3**	**4.4 ± 0.5**	**3.4 ± 1.8**	**5.1 ± 0.3**
